# Parkinson’s Disease: Pathophysiology, Treatment Strategies, Wellness Approaches, and Obstacles/Paths Forward

**DOI:** 10.3390/neurosci7040093

**Published:** 2026-08-21

**Authors:** Frank C. Church, Jill C. Rau, Stefania Brotini

**Affiliations:** 1 Department of Pathology and Laboratory Medicine, School of Medicine, The University of North Carolina at Chapel Hill, Chapel Hill, NC 27599, USA; 2 Department of Neurology, Bob Bové Neuroscience Institute at HonorHealth, Scottsdale, AZ 85251, USA; jillrau@honorhealth.com; 3 Movement Disorders Center, Department of Neurosciences, AUSL Toscana Centro, San Giuseppe Hospital, 50053 Empoli, Italy; stefania.brotini@uslcentro.toscana.it

**Keywords:** Parkinson’s disease, neurodegenerative disorder, dopamine, motor and non-motor symptoms, exercise, sleeping disorders, health and wellness, quality of life

## Abstract

Parkinson’s disease (PD) is a neurodegenerative disorder caused by the loss of dopaminergic neurons. Its symptoms affect both motor functions—such as bradykinesia, rigidity, resting tremor, and postural instability—and non-motor functions, including neuropsychiatric issues, sleep disorders, and constipation. Drawing on the literature, the following topics were developed and presented: Pathophysiology; Treatment; Neuropsychiatric and Cognitive Function; Sleep Challenges; Exercise and Movement; Striving for Wellness; and Obstacles/Paths Forward. These seven categories form the foundation of this review, which centers on four main areas related to PD: dopamine biology and pathophysiology; management of key motor and non-motor symptoms; strategies for achieving wellness; and unresolved issues in PD. The complexity of PD’s clinical features is highlighted by describing patient–healthcare provider interactions, elucidating disease mechanisms, managing motor and non-motor symptoms, highlighting the benefits of exercise, emphasizing the importance of sleep, addressing mental health challenges and cognitive changes, fostering a hopeful and resilient mindset, potentially slowing disease progression, treating comorbidities, and promoting overall wellness. Although there have been tremendous advances in the basic science and clinical management of PD in recent years, PD remains incurable. Thus, we describe several roadblocks that have hindered progress in understanding PD. Only by integrating historical insights with the latest PD advances can we acquire the knowledge needed to change the disease’s trajectory. Finally, this review aims to provide a meaningful summary for healthcare professionals (and students) caring for patients with PD.

## 1. Introduction

### 1.1. Neurodegenerative Diseases

Neurodegenerative diseases involve the death of neurons caused by ongoing dysfunction, leading to motor (movement) problems, cognitive decline (dementia), or both [1,2,3]. Alzheimer’s disease and Parkinson’s disease (PD) are the two most prevalent neurodegenerative disorders, respectively. In 1817, James Parkinson published “An Essay On The Shaking Palsy,” which was the first medical document to describe PD in detail [4,5]. Parkinson named this disorder shaking palsy (paralysis agitans).

### 1.2. Parkinson’s Disease (PD)

PD symptoms stem from the gradual loss of dopamine neurons in the substantia nigra pars compacta [4,6,7,8,9], a process that develops over years, making diagnosis difficult [4,8]. Historically, it has been considered a motor disorder characterized by four main symptoms: bradykinesia, rigidity, postural instability, and tremor (Table S1) [10,11,12,13,14,15]. However, there are many non-motor symptoms, such as constipation, urinary issues, depression, psychosis, and sleep disturbances, that often accompany and precede motor symptoms by decades (Table S1).

### 1.3. Etiology of PD

PD affects about 1% of adults over 60, mostly sporadically caused by age, α-synuclein (α-Syn) buildup, genetics, neuroinflammation, immune issues, mitochondrial problems, oxidative stress, and environmental toxins [16,17]. Young-onset PD is generally genetic. The complex causes of sporadic PD require a multifaceted treatment to slow or halt progression. PD is a chronic, progressive disorder [7,8,18,19,20,21,22].

### 1.4. PD Pandemic

In 2018, it was suggested that there was a pandemic of rising PD cases [23,24], partly due to exposure to toxins like pesticides and solvents [25], and driven by aging populations [25], since age is a key risk factor [26]. Future increases may also be linked to SARS-CoV-2 infection during COVID-19 [27]. Trends from 1990 to 2021 show PD burden grew over 32 years [28,29], with projections that PD will be a major health issue by 2050 [30].

### 1.5. Medical Care Treatment Trend for PD

Using 2019 Medicare data, a recent study highlighted care access and use among individuals with PD [31]. Only 9% received care from movement disorder specialists, 51% from general neurologists, 29% from primary care physicians, and 29% had no physician. The study also found 80–90% of patients did not see therapists, and over 95% of those with depression lacked mental health support [31]. Another study by the International PD Foundation emphasized the importance of rehabilitation in PD care [32]. These findings underscore the need for a coordinated, multidisciplinary approach to improve PD care [33].

### 1.6. Elements of This PD Review

**Figure 1:**
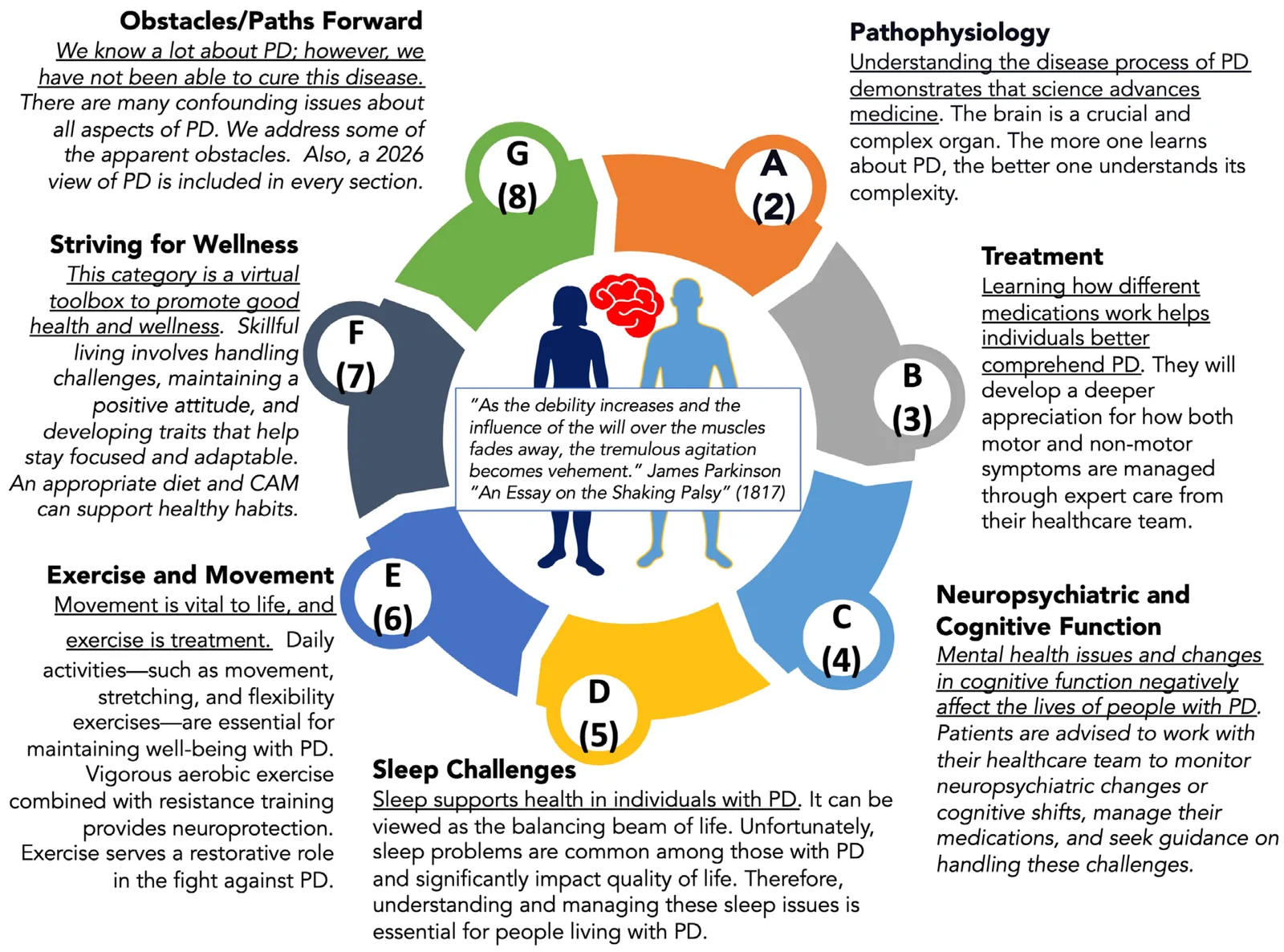
An overview of the categories used in this PD review (The number in parentheses next to the letter indicates the text section number for that category in the paper). Each category includes a brief description explaining how someone could benefit from understanding the material within it. The literature searches were performed with PubMed and Google Scholar with search terms in various combinations with “Parkinson(’s) disease”: “neurodegeneration”, “neuropathology”, “dopamine”, “neurotransmitter”, “neuromodulator”, “basal ganglia”, “substantia nigra”, “nigrostriatal pathway”, “mesocortical pathway”, “mesolimbic pathway”, “dopaminergic neurons”, “neuroinflammation”, “(non-)motor symptoms”, “orthostatic hypotension”, “constipation”, “executive function(s)”, “fatigue”, “chronic pain”, aerobic exercise”, “resistance training”, “dietary inflammatory index”, “positive”, “grateful”, “neuropsychiatric”, “psychosis”, “dementia”, “impulse control disorder”, “apathy”, “anxiety”, “depression”, “cognitive”, “skillful living”, “character traits”, “hope”, “persistence”, “insomnia”, “excessive daytime sleepiness”, “circadian dysfunction”, “obstructive sleep apnea”, “rapid eye movement (REM) sleep behavior disorder (RBD), “α-synuclein”; “synucleinopathy”; and “proteinopathy”. From these literature searches, we compiled review papers and primary research on the topic and assessed the historical information against what is known today. The major categories were formed from the literature searches, and the topics presented were chosen by the authors; thus, this is a narrative review.

With an aging population, a potential pandemic, and a shortage of general neurologists and movement disorder specialists, a major challenge arises. Healthcare personnel need both historical and current insights to stay informed about this complex disease. Our goal is to provide PD education for healthcare students and professionals. Figure 1 presents the specific areas covered in this narrative review.

## 2. Pathophysiology

### 2.1. Dopamine, Dopamine Receptors, and the Nigrostriatal Pathway

Dopamine is an essential neurotransmitter derived from catecholamines, with the formula C_8_H_11_NO_2_, and is related to norepinephrine and epinephrine [34,35,36,37,38]. It functions as a key chemical messenger between neural networks involved in movement, motivation, reward, and cognitive processes [35,37,38,39]. Dopamine is produced in the substantia nigra pars compacta and ventral tegmental area (VTA), both located in the midbrain. Dopamine is critical for proper brain function [37,38,40,41].

Dopamine binds to five G protein-coupled receptors (GPCRs) [42], grouped as D1-like (D1, D5) and D2-like (D2, D3, D4) dopamine receptors [37,38]. D1-like dopamine receptor activation stimulates adenylyl cyclase via G_as/olf_, increasing cAMP and activating PKA, which phosphorylates DARPP-32 and transforms it into a potent inhibitor of Protein Phosphatase-1 (PP-1). This reduces Na^+^ and K^+^ channel activity but opens L-type Ca^2+^ channels, boosting NMDA responses and neuronal excitation [39,40]. When dopamine binds to D2-like dopamine receptors, this activates G_ai/o_ proteins to inhibit adenylyl cyclase, decrease PKA activity, open K^+^ channels, close Ca^2+^ channels, and inhibit neuronal activity [40,41]. Both D1-like and D2-like dopamine receptors trigger the up-regulation and activation of tropomyosin receptor kinase B (TrkB), the main BDNF receptor [41].

Levodopa does not bind to dopamine receptors to trigger a therapeutic response. It acts much like a “Trojan horse” because it can cross the blood–brain barrier and, once inside, is converted into dopamine by the enzyme DOPA decarboxylase [43]. Dopamine binding to the D1-like dopamine receptors (Excitatory Effect) activates the direct pathway in the striatum. These excited neurons release GABA onto the internal globus pallidus and substantia nigra pars reticulata, which then disinhibits the thalamus and thereby promotes movement [44,45]. Dopamine binding to the D2-like dopamine receptors (Inhibitory Effect) inhibits the indirect pathway of the striatum (the pathway that normally “brakes” movement), thereby unlocking motor functions [44,45].

The basal ganglia control movement through these excitatory and inhibitory mechanisms [37,38]. The nigrostriatal pathway involves dopamine from the substantia nigra pars compacta acting on D1-like dopamine receptors to facilitate the direct pathway and enhance movement. Conversely, dopamine acting on D2-like dopamine receptors inhibits the indirect pathway, which also facilitates motor activity [37,38,41,46,47,48,49].

The loss of dopaminergic neurons in the substantia nigra alters the nigrostriatal pathway [50]. Reduced dopamine synthesis results in less positive motor action and more negative motor inhibition, leading to the hypokinetic dysfunction usually found in people (person)-with-Parkinson’s disease (PwP).

### 2.2. Role of Dopaminergic Pathways in Developing PD Motor and Non-Motor Symptoms

**Figure 2:**
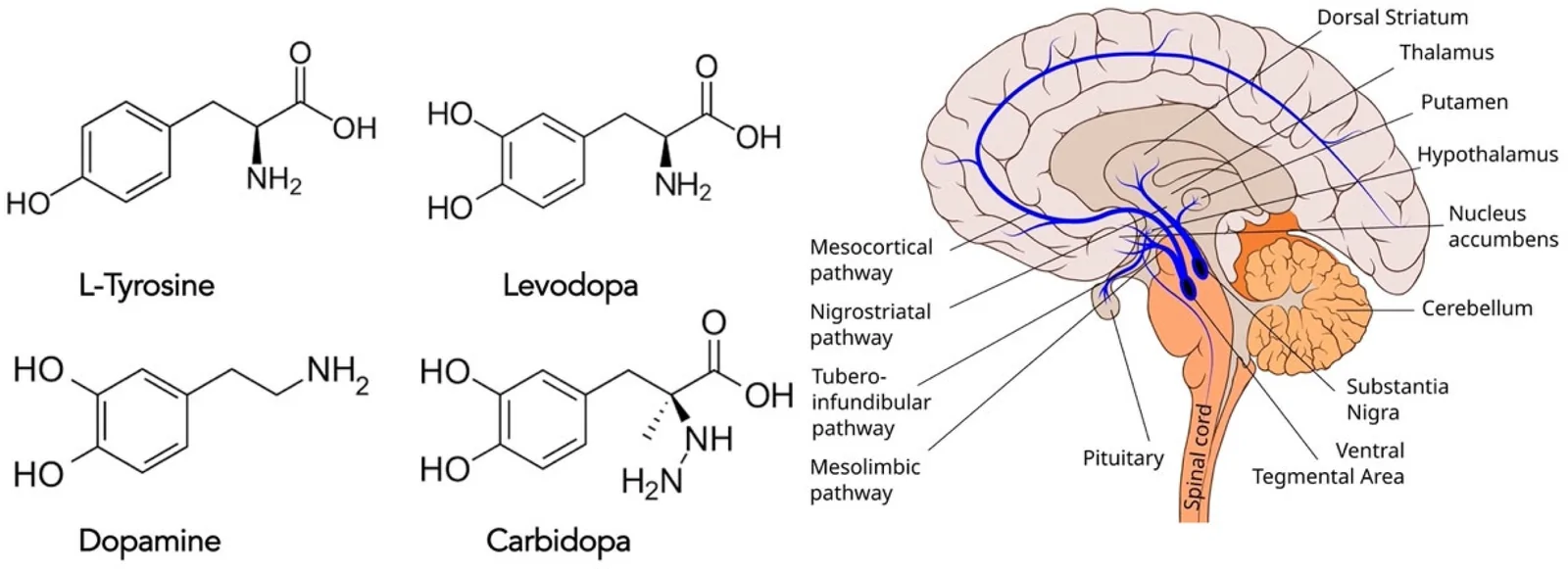
Chemical structures of dopamine and its precursor tyrosine, and the major drugs used to treat PD, levodopa and carbidopa (**left panel**), and the main dopaminergic pathways of the human brain (from User:Slashme; Patrick J. Lynch; User:Fvasconcellos, CC BY-SA 4.0 <https://creativecommons.org/licenses/by-sa/4.0>, via Wikimedia Commons) (**right panel**).

Several factors contribute to dopaminergic neuron death in PD (described in the Introduction). This initial process is slow, typically measured in years, as symptoms usually appear only after more than 50% of neurons have been lost. Figure 2 illustrates various aspects of dopamine, including the relationship between the structures involved in its synthesis and the compounds used to treat a PwP (left panel, Figure 2). Additionally, the brain cross-section schematic shows the locations of pathways that interact with dopamine and facilitate its many biological actions (right panel, Figure 2).

**Figure 3:**
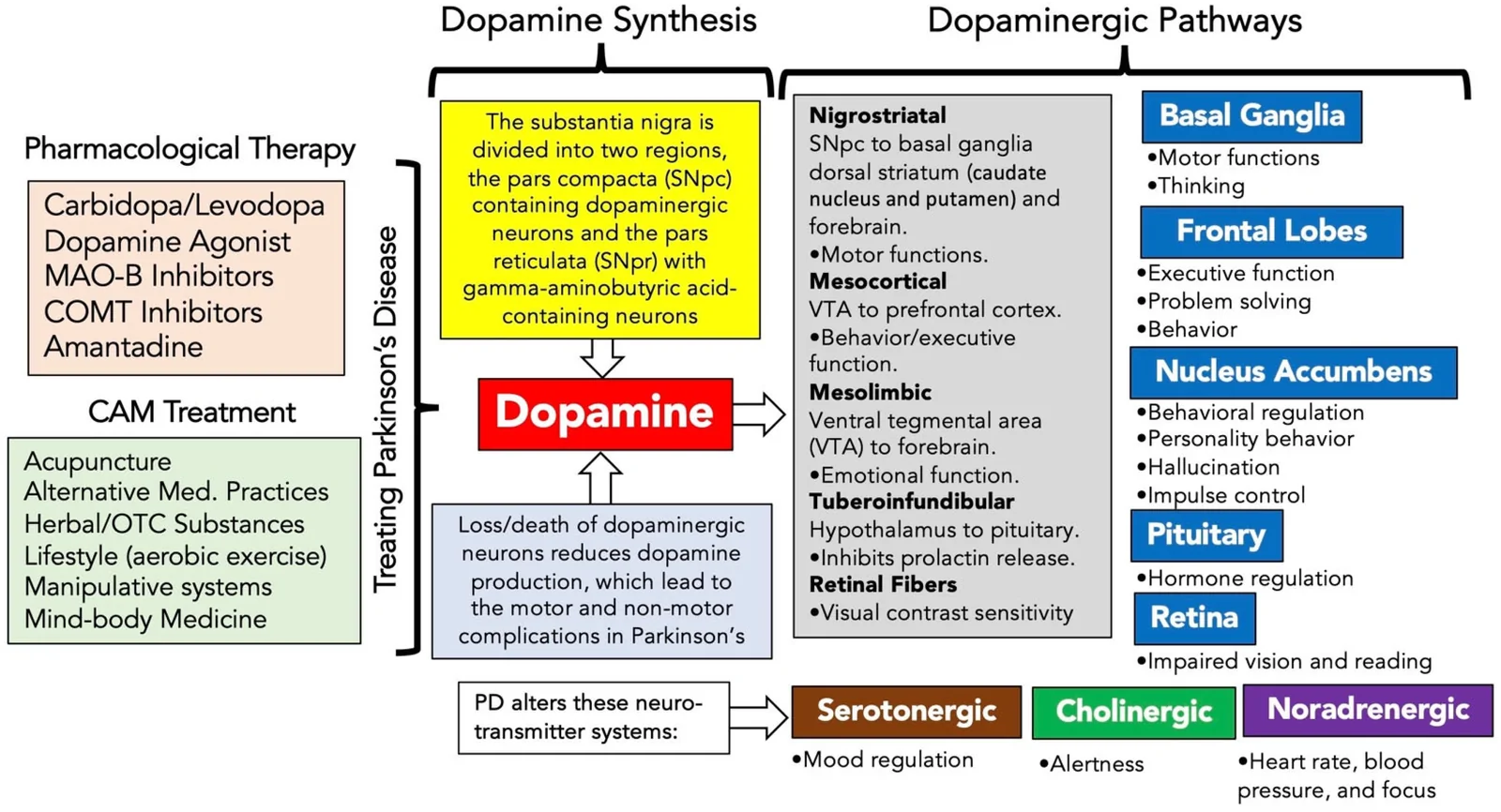
Overview of dopamine’s physiological pathways and roles. This figure is modified from [57].

Various dopamine pathways are shown in Figure 3 [36,51,52,53]. Dopamine influences motor and non-motor functions, including cognition, mood, sleep, sensory processes, and autonomic systems. It acts as a neurotransmitter and neuromodulator, affecting other neurotransmitters [54,55,56]. Dopamine loss impacts both functions (Figure 2 and Figure 3).

To enhance the clinical relevance of Figure 2 and Figure 3, Table 1 presents the clinical consequences of these pathological changes as PD progresses.

### 2.3. Dopamine’s Role as a Neuromodulator in Systems Neuroscience

Systems neuroscience investigates how extensive networks of brain cells collaborate to generate senses, thoughts, movements, and behaviors [58,59,60]. It broadens the scope from single cells to whole brain circuits. Systems neuroscience characterizes dopamine as a flexible neuromodulator that signals reward prediction errors, incentive salience (“prompting organisms to seek out goals and stimuli deemed valuable for survival or well-being” [61]), and motivational states across neural circuits [62]. Contemporary theories have yielded several perspectives on dopamine and dopaminergic neurons: (i) Reward Prediction Error, in which midbrain dopamine neurons fire in bursts to serve as a teaching signal for reinforcement learning; (ii) Beyond Valence, calcium imaging and optogenetics reveal that, movement kinematics, or alerting salience rather than pure reward; and (iii) Modulation of Neuroplasticity, as dopamine binding to D1-like and D2-like receptors regulates long-term potentiation and depression in target areas such as the striatum and hippocampus, setting thresholds for structural and synaptic changes [62,63,64]. Therefore, recognizing that the dopamine neurons in the VTA and substantia nigra are functionally diverse—some encoding aversive stimuli (the brain process of detecting, processing, and learning from negative, painful, or threatening events [65])—is essential, given their roles not only in movement but also as neuromodulators [39].

### 2.4. Pathology of PD

One of the more unusual features of PD is the presence of Lewy bodies [17,66,67,68,69]. Fritz Heinrich Lewy was a German neurologist who, in 1912, described unusual cytoplasmic deposits (bodies) in the substantia nigra [70]. We now know that the primary component of Lewy bodies is a protein called α-synuclein (α-syn). Significantly, α-syn in the Lewy bodies is aggregated (or denatured) and not in its native globular (or soluble) form. As Lewy bodies accumulate in dopaminergic neurons, these neurons die by diverse mechanisms [71].

### 2.5. Role of α-Synuclein in PD

Finding α-syn in Lewy bodies was interesting, but distinguishing it as part of the disease process rather than a biomarker was more important [72,73,74]. Identifying genetic mutations in α-syn in PD supports a pathophysiological role [75,76]. The native form of α-syn is found at presynaptic sites, involved in neurotransmitter release and reuptake, and regulates synaptic vesicle pools and trafficking [77]. It modulates dopamine storage in vesicles by influencing VMAT2 activity in neurotransmitter neurons [78].

α-Syn is present in neurons, blood cells, and tissues, existing as both monomers and tetramers [79,80]. Pathological α-syn arises from aggregates formed through seeding within dopaminergic neurons [81]. Its aggregation impairs mitochondrial function and may contribute to neuron degeneration. Oxidative stress damages cells by suppressing the ubiquitin-proteasome system, leading to neuronal death. Studies suggest that α-syn aggregation contributes to neuroinflammation by activating microglia and promoting neuronal death [82,83]. Whether α-syn causes neurodegeneration or is a response remains debated; initial deposits might be protective. Establishing causality in PD is complex.

Causal hypothesis: Many studies identify α-syn accumulation as a key factor, with aggregates and Lewy bodies damaging neurons and mitochondria and triggering microglial activation, leading to neuroinflammation and degeneration.

Adaptive response hypothesis: Some believe deposits may form after or as a defense against toxic soluble forms because post-mortem studies cannot determine the sequence.

Finally, α-syn is a key PD drug target, with strategies including monoclonal antibody immunotherapies, aggregation inhibitors, and regulation of immune responses or protein clearance.

### 2.6. Genetic Factors of PD

PD has a genetic component, with mutations accounting for 10–15% of cases, mainly early-onset, often with a family history, indicating genetic links [16,84,85,86]. Most cases are idiopathic and affect those over 60–65 [84]. Research shows that both monogenic forms and risk factors influence PD. Understanding these variants helps identify therapeutic targets [6,87]. Genes linked to PD include [gene name (historical designation)—inheritance pattern; and role in PD]:

*SNCA* (PARK1)—α-syn; autosomal dominant; Lewy body formation [6].

*LRRK2* (PARK8)—G2019S mutation; autosomal dominant; late-onset familial form [87].

*PRKN* (Parkin, PARK2)—Autosomal recessive; early-onset (<40 years) [88].

*PINK1* (PARK6)—Autosomal recessive; mitochondrial function [89].

*DJ-1* (PARK7)—Autosomal recessive; rare juvenile forms [90].

*UCHL1* (PARK5)—Autosomal dominant; very rare [87].

*MAPT*—Risk factor; influences cognitive decline [91].

*APOE*—Risk factor; affects disease progression [91].

*GBA1*—Major; increases PD risk up to 5–6 times [92,93].

*MS4A4E*, *DKKL1*, *MPV17L2*, *MIR499A*, *4AGAP2*, *CLECL1*, *CLEC16A*, *MIR196A2*, *IL7R*, *INPP5D*, *ZSWIM4*—Newly identified genes [91].

### 2.7. Gut–Brain Axis of PD

Many PwP exhibit gastrointestinal (GI) tract problems before motor symptoms. The so-called gut–brain axis in PD suggests that signals from the gut contribute to its pathogenesis [94,95,96,97,98,99]. Furthermore, substantial evidence shows that the gut microbiome is altered in PwP compared with age-matched individuals without PD. Moreover, α-syn aggregation, characteristic of dopaminergic neurons in PwP, may also initiate in the gut and subsequently spread to the brain via the vagus nerve [100]. There is substantial evidence that many PwP will have one or more of these GI tract disturbances: dysphagia, sialorrhea, bloating, nausea, vomiting, gastroparesis, and constipation [101].

### 2.8. Current Overview of PD Pathophysiology and Recent Advances

Historically, the PD model was a dopamine deficiency in the substantia nigra pars compacta, which well describes the motor symptoms. The up-to-date view of PD pathophysiology is that it is a complex, multi-neurotransmitter, multi-system neurodegenerative disease [102,103,104], which involves other neurotransmitters [105], the almost “prion-like” spread of α-syn throughout the central and peripheral nervous systems [106], loss of energy production from mitochondrial dysfunction [107], oxidative stress and neuroinflammation [108], genetic mutations combined with environmental risk factors [109,110], and the development of the disease in the gut, which communicates through the vagus nerve [106].

Basic Research: Synucleinopathies are a group of neurodegenerative diseases characterized by the abnormal accumulation of the protein α-syn [68]. A major focus of PD research is understanding what causes the abnormal buildup of α-syn. Recently, PD was described as a fatty acidopathy [111]. This work explains PD as a condition characterized by fatty acid dyshomeostasis, an imbalance in fatty acid composition within cells or systems [111]. The brain is rich in fatty acids, and their dysfunction may contribute to abnormal interactions between α-syn and lipids, potentially promoting α-syn aggregation and contributing to PD development [111].

In other research, a series of helix-constrained peptides based on the α-syn sequence was tested to determine whether aggregation into toxic oligomers could be prevented [112]. The most effective peptide, which formed an α-helix, increased soluble α-syn levels, indicating that it inhibited aggregation [112]. Using the NL5901 *C. elegans* model of PD, the candidate peptide rescued the progressive loss of motility, demonstrating it could cross biological membranes and effectively inhibit α-syn aggregation. Overall, this work provides an opportunity to develop a peptide-based strategy for treating PD [112].

## 3. Treatment

### 3.1. Making the Diagnosis of PD

The diagnosis of PD is based on clinical features, including medical history and physical examination [113,114]. Among all typical motor symptoms, bradykinesia correlates most strongly with dopamine deficiency [115]. To confirm a patient’s diagnosis of PD, two cardinal motor signs are required: typically, bradykinesia and either rigidity or tremor. Since several non-motor symptoms may precede motor symptoms, a careful history can reveal this pattern, helping the physician make a more accurate diagnosis of PD [113,114].

### 3.2. Stages and Progression of PD

Recognize that each PwP is unique in symptom burden and progression rate [116,117,118], necessitating a PD staging system. Such a system standardizes disease assessment—symptom spread and progression—enabling healthcare professionals to communicate effectively. It aids neurologists in treatment planning and in predicting prognosis. PD progression varies: some PwP progress slowly over years, others rapidly in a few years. The first staging tool, the Hoehn and Yahr (HY) scale [119], classifies Parkinson’s disease based on motor symptoms. Other staging tools include MDS-UPDRS, a revision of the Unified Parkinson’s Disease Rating Scale (UPDRS) from the 1980s, assessing motor and non-motor symptoms, daily activities, and motor complications [120]; more recently, research focuses on biomarkers, like those used for cancer, to classify PD based on disease biology [121]. The MDS-UPDRS is the gold standard for clinical evaluation, staging, and monitoring of disease severity.

### 3.3. What Is Atypical Parkinsonism?

Parkinsonism is a broad term for disorders with symptoms like PD, caused by various factors [121,122,123,124,125]. Secondary Parkinsonism includes medication side effects, head trauma, metabolic diseases, toxins, and neurological disorders from external factors like heavy metals or infections. PD is a specific type of Parkinsonism. Diagnosis relies on recognizing symptom patterns, but this is challenging. Primary Parkinsonian syndromes, or “atypical Parkinsonisms,” are distinct degenerative disorders that progress faster and cause earlier morbidity. Key features of atypical Parkinsonism include Parkinsonian symptoms, but with little or no response to levodopa therapy.

Faster progression compared to classic PD.

Additional signs not typical of idiopathic PD include early balance disturbances, cognitive impairment or dementia, autonomic dysfunctions (blood pressure, bladder), and abnormal eye movements.

Some atypical Parkinsonisms are [122,123,124,125,126]:

Multiple system atrophy (MSA) results in early dysautonomia and a rapid need for a wheelchair.

Progressive supranuclear palsy (PSP) leads to early falls, swallowing problems, and gaze palsy.

Corticobasal degeneration (CBD) results in patients having asymmetric symptoms, limb ataxia, apraxia, alien limb, and sensory issues.

Frontotemporal dementia (FTD) involves cognitive decline and Parkinsonian symptoms within 1–3 years.

Dementia with Lewy Bodies (DLB) combines features of movement disorders and neurodegenerative dementias. It presents with Parkinsonism symptoms such as rigidity, bradykinesia, and postural instability, sharing many motor traits with PD, and is a synucleinopathy. DLB is not classified as atypical Parkinsonism, such as Parkinson’s Disease Dementia (PDD). The key difference is the “one-year rule”: in DLB, dementia occurs before or within a year of motor symptoms, whereas in PDD, it develops at least a year after PD. Lewy bodies are found in cortical neurons in DLB and in the substantia nigra in PDD.

### 3.4. Differential Diagnosis of PD

As described above, the diagnosis of PD refers to the criteria used to identify PD (such as gut–brain axis biomarkers, early dopamine loss, or clinical staging). From there, the differential diagnosis in PD is crucial for distinguishing idiopathic disease from atypical parkinsonian syndromes (e.g., PSP or MSA) or essential tremor, relying precisely on the distinct alterations in the neurochemical networks we have discussed.

### 3.5. Treating the Motor Symptoms of PD

The leading causes of death among adults are heart disease, cancer, accidents, COVID-19, and stroke, not PD [127]. While PD can lead to illness and death, managing comorbidities is complex and underscores the importance of a diverse care team to deliver comprehensive treatment.

Levodopa has been the main PD treatment for over 50 years [8,128,129]. Levodopa does not slow or cure PD but remains the primary medication for motor symptoms.

**Figure 4:**
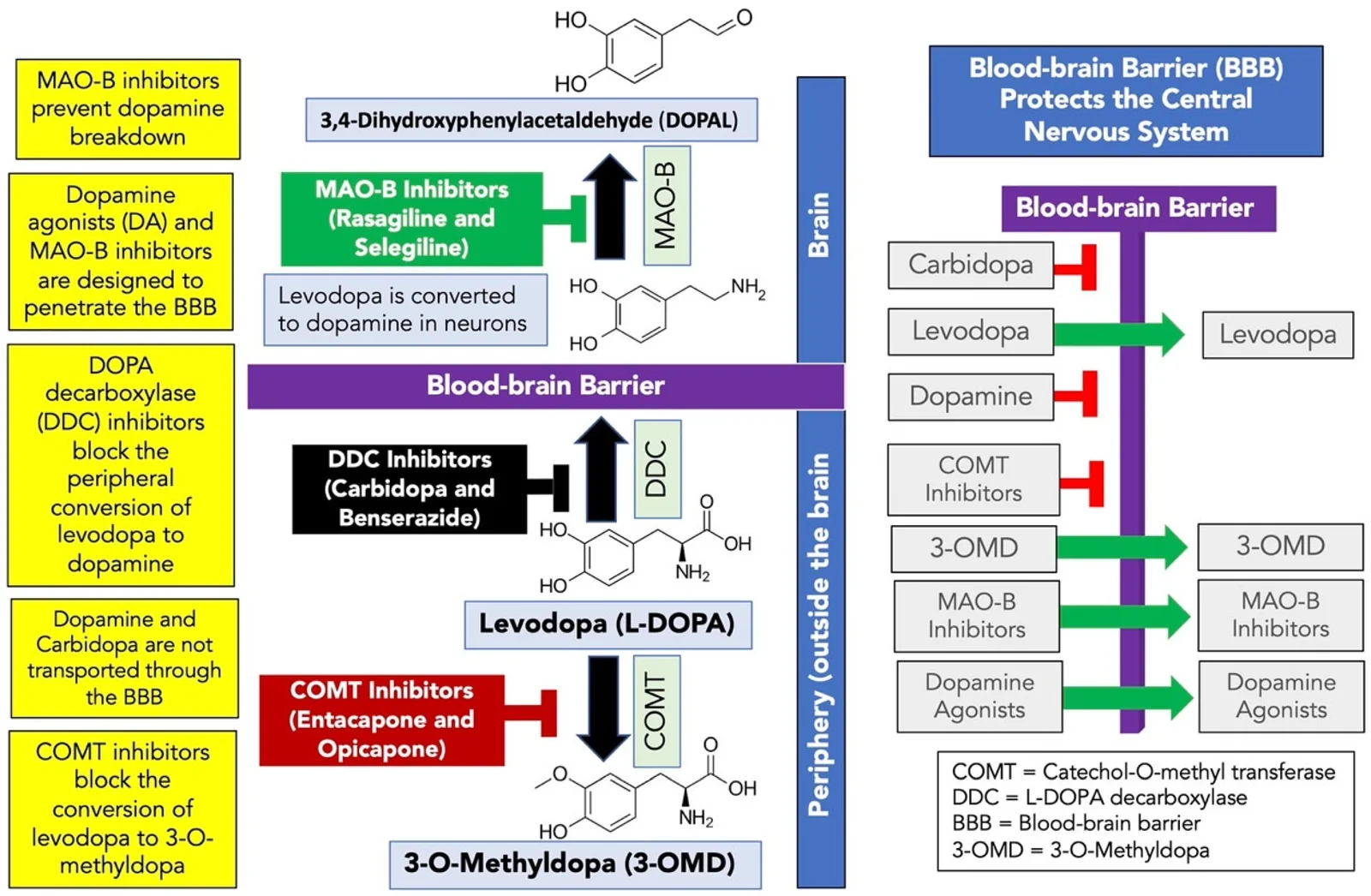
Function of various drugs to regulate the motor symptoms of PD.

Dopamine, derived from tyrosine [130], is produced when the enzyme tyrosine hydroxylase converts L-tyrosine into L-DOPA (or levodopa), which is then decarboxylated to dopamine. Treating PD motor symptoms involves (i) delivering levodopa, (ii) using dopamine mimics like dopamine agonists, (iii) preserving dopamine levels with MAO-B inhibitors, or (iv) maintaining circulating levodopa with COMT inhibitors (Figure 4).

Combining levodopa with carbidopa (or benserazide) offers the most relief from PD symptoms with minimal side effects [131,132]. These drugs prevent peripheral conversion of levodopa to dopamine, increasing CNS delivery [133]. The blood–brain barrier allows levodopa entry but blocks the entry of dopamine and carbidopa (Figure 4). Various formulations exist (Table S2), including Duodopa, which provides continuous gel infusion [8]. Inhaled levodopa (Inbrija) and subcutaneous infusion (Vyalev) are also available. Common side effects include dyskinesia and motor fluctuations, such as wearing-off and on–off phenomena [8].

Levodopa-containing compounds: Several substances are used, beginning with the simplest formulation of levodopa-carbidopa at a 4:1 ratio. Recent additions to the carbidopa-levodopa derivative class are Rytary and Crexont, extended-release formulations with significantly longer plasma half-lives to minimize on-off fluctuations (Table S2).

Dopamine agonists: These drugs bind to dopamine receptors to compensate for decreased dopamine levels. For many PwP, a dopamine agonist may initially be used to treat their PD. Some dopamine agonists can lead to troublesome side effects, such as promoting impulse control disorders [134] (Table S2).

Monoamine oxidase B (MAO-B) inhibitors: Dopamine is broken down in the brain by the enzyme called monoamine oxidase B. MAO-B inhibitors are used to maintain dopamine levels in the CNS, thereby increasing its availability (Table S2). Interestingly, safinamide (Xadago) has MAO-B inhibitory effects, inhibits glutamate release, and blocks calcium and sodium channel activities, which may offer adjunctive therapy for both motor and non-motor symptoms of PD.

Catechol-O-methyl transferase (COMT) inhibitors: COMT inhibitors prevent the conversion of levodopa to 3-O-methyldopa, thereby prolonging levodopa’s half-life. PwP experiencing motor fluctuations from levodopa have seen their symptoms improve with the addition of a COMT inhibitor (Table S2).

Amantadine: Amantadine can control tremors. Thus, amantadine may help manage prominent tremors or PwP with dyskinesia from long-term levodopa use (Table S2).

Anticholinergics: Medications mainly used to treat tremor, but nowadays, they are sometimes used only in younger patients (<65 years old) (Table S2).

### 3.6. Motor Complications in PD: Genesis and Pathophysiology

After several years of levodopa replacement therapy (typically within 5–10 years of treatment initiation in 80% of patients), the drug’s efficacy shifts from a stable, continuous response (the so-called “honeymoon phase”) to an unstable, fragmented one [135]. This phenomenon manifests as motor complications, which are broadly divided into two main categories:

Motor Fluctuations: Daily oscillations in therapeutic effect that can exist in two different phases: wearing-off, which is the re-emergence of motor symptoms before the next scheduled dose of levodopa; and unpredictable “ON/OFF” phases that result in sudden shifts from a state of good mobility (ON) to severe motor immobility (OFF), occurring independently of the medication schedule [136].

Dyskinesias: Abnormal involuntary movements (choreic or dystonic) that predominantly manifest during peak plasma concentrations of the drug (peak-dose dyskinesias).

The pathogenesis of motor complications is multifactorial, resulting from the complex interplay between neurodegenerative progression and levodopa pharmacokinetics [137]. In the early stages of the disease, surviving dopaminergic neurons can still take up exogenous levodopa, convert it into dopamine, store it in vesicles, and release it in a tonic, constant fashion into the synaptic cleft. As neurodegeneration advances, the loss of these presynaptic terminals deprives the striatum of its storage capacity. Striatal dopamine levels thus become entirely dependent on the drug’s fluctuating plasma concentrations. Levodopa has a short plasma half-life (approximately 60–90 min). Due to the loss of presynaptic buffering, post-synaptic striatal dopamine receptors are subjected to pulsatile stimulation (rapid peaks and troughs) rather than to physiological tonic, continuous stimulation [138].

Pulsatile stimulation deeply alters intracellular signaling pathways within striatal medium spiny neurons. A prominent dysregulation of glutamate receptors (specifically NMDA and AMPA) occurs, leading to maladaptive synaptic plasticity (akin to corrupted long-term mechanisms) [139]. This induces hyperactivity of the basal ganglia direct pathway and mistuning of motor circuits, which clinically translates into dyskinesias.

### 3.7. Neuromodulation Therapies in PD

Neuromodulation, targeting brain networks, is an emerging field. With a greater understanding of both neural circuit anatomy and function, neuromodulation therapy has advanced dramatically and now includes symptom-specific techniques. Neuromodulation techniques are divided into invasive and non-invasive approaches, with increasingly personalized strategies:

Deep Brain Stimulation (DBS) surgery is usually the next treatment option for advanced PD [131,132]. DBS typically targets the thalamus, globus pallidus, or subthalamic nucleus. DBS reduces movement-related symptoms, such as tremors, stiffness, and slowness. Like other PD treatments, DBS is not curative [133]; however, a PwP who undergoes DBS may require much lower doses of carbidopa-levodopa. Interestingly, both dopamine (using levodopa/carbidopa) and subthalamic DBS can lessen akinesia (impaired action initiation). This suggests that similar neural circuit synaptic connectivity (i.e., connectomics) underlies both approaches and opens paths to better understanding how DBS treats PD.

Adaptive DBS (aDBS) adjusts stimulation amplitude in real time based on the patient’s brain signals, improving efficacy and reducing side effects [140].

Remotely programmable neurostimulators allow therapy adjustments at a distance, reducing travel and improving continuity of care.

Noninvasive neuromodulation techniques:

- Transcranial Magnetic Stimulation (TMS) modulates cortical activity to improve motor and nonmotor symptoms.
- Transcranial Direct Current Stimulation (tDCS) and Alternating Current Stimulation (tACS) influence neuronal excitability and synaptic plasticity.
- Photobiomodulation uses light to modulate brain activity.

Emerging approaches:

Photothermal nanoparticles and laser use wireless stimulation of dopaminergic neurons, with selective targeting and reduction in pathological α-syn aggregates; currently experimental in animal models.

AI-based DBS algorithms predict brain activity and proactively adapt stimulation parameters.

### 3.8. Options for Treating Motor Symptoms

The initial PD treatment protocol varies based on factors such as age, employment, lifestyle, motor symptom severity, and non-motor symptoms [141,142]. As the disease progresses, treatment is adjusted to preserve quality of life.

Tremor: Most patients respond to levodopa-containing medications and dopamine agonists (see potential side-effect of impulse control disorders). Adjunctive substances may be used to manage the on-off fluctuations during disease progression, and in advanced PD, DBS surgery can be beneficial.

Rigidity: Levodopa-containing substances help maintain dopamine levels, which reduce stiffness. Dopamine agonists have a similar effect on decreasing rigidity. Stretching and range-of-motion exercises (e.g., PWR!Moves), strengthening exercises (e.g., Rock Steady Boxing), dancing (e.g., Dance for Parkinson’s Disease), and yoga and tai chi help reduce stiffness [20].

Bradykinesia: As with rigidity, the same combination of medication and exercise/physical therapy/dance programs shown above helps treat bradykinesia.

Postural instability: The exercise/physical therapy/dance/yoga/tai chi programs above help treat postural instability, while levodopa is only effective in the early to mid-stages of PD.

Gait disturbances: A combination of medication (levodopa-containing compounds), specific exercises with physical therapists, and sensory-based work can improve gait problems.

Speech problems: The overall goal of specific voice therapy programs (e.g., LSVT LOUD and SPEAK OUT!) is to improve clarity and loudness by strengthening vocal muscles.

Handwriting changes: This is primarily achieved by using a thick-gripped or weighted pen, engaging in specific writing exercises, and writing mainly during the ‘on’ phase of levodopa therapy.

Facial masking: Treatment involving medications that boost dopamine and collaborating with physical and occupational therapists to improve facial muscle function.

Dystonia: Medications that increase dopamine levels can help improve dystonia, a condition characterized by involuntary, sustained muscle contractions that cause twisting, repetitive movements, and abnormal postures. Botulinum toxin injections can block muscle contractions, such as neck twisting or torticollis; anticholinergics can inhibit acetylcholine; and physical or occupational therapists can help design specific exercises to reduce dystonia.

Dyskinesia: Dyskinesia is a complication of long-term carbidopa-levodopa use and higher doses, affecting most PwP, who experience uncontrolled movements or fidgeting [12,143]. It can be more bothersome than motor symptoms. Treatment options include adjusting the levodopa dose or timing to manage symptoms, adding amantadine (e.g., extended-release Gocovri) [143], or switching to extended-release carbidopa-levodopa formulations (Rytary, Crexont, or Duopa) to enhance stability. Emerging evidence suggests that omega-3 fish oils containing DHA may also help reduce dyskinesia [144,145].

### 3.9. Freezing of Gait (FOG)

FOG is a common and complex symptom in PD, involving both motor and non-motor factors. It occurs when PD patients feel as if their feet are glued to the floor, especially at doorways, during turns, in narrow spaces, changing surfaces, in darkness, or under stress [146,147,148,149]. FOG increases fall risk and lowers quality of life, often occurring during the “off” phase of dopaminergic medication, suggesting a motor component. Therefore, neurologists should focus on optimizing dopaminergic therapy. FOG is also linked to impaired neural networks related to cognition, attention, visuospatial skills, and sleep. Understanding these interactions could lead to new treatments. Meanwhile, patients should seek physical therapy strategies to manage FOG.

### 3.10. Disruption of the Autonomic Nervous System (ANS) Leads to Several Non-Motor Symptoms

PD is not a simple, uniform disease. Motor symptoms mainly respond to dopamine therapy, while many non-motor symptoms do not. Here, we outline treatment for common non-motor symptoms related to ANS dysfunction.

The ANS controls involuntary functions such as heart rate, digestion, urination, and sweating [150], and PD can affect it, causing non-motor symptoms. α-Syn accumulation and genetic/environmental factors contribute to ANS dysfunction in PD [151], reducing quality of life [152]. Dysphagia in PD arises from motor and non-motor issues, raising aspiration risk [153] and involves the muscles, the ANS, and GI system dysfunction. Treatment includes levodopa, dopamine agonists, dietary modifications, speech therapy, and possibly strengthening expiratory muscles with devices like EMST 150.

Sialorrhea: Excessive drooling occurs when the swallowing reflex is slowed, leading to increased saliva buildup [154]. Treatment options for sialorrhea include atropine drops, botulinum toxin injections, glycopyrrolate, and a scopolamine patch (Table S3).

Constipation: A common gastrointestinal problem in PD [155]. In addition to drinking plenty of fluids, following a good diet, and, if necessary, taking a dietary fiber supplement, several treatment options for constipation are available, including Lubipostone, polyethylene glycol 3500, and Linaclotide (Table S3).

Orthostatic hypotension: It can happen in PwP when moving from lying down or sitting to standing [156]. A careful history is needed to treat orthostatic hypotension, including adjusting motor-specific medications, wearing support stockings, increasing fluid intake, and then switching to the medications listed in Table S3.

Urinary urgency: Loss of bladder control caused by urinary urgency or frequency is a relatively common symptom in PD [157]. Urinary urgency at night can significantly impact the quality of life for individuals with PD. After ruling out urinary tract infections or benign prostatic hyperplasia in men, managing urinary urgency can be done with the medications listed in Table S3.

### 3.11. Chronic Pain

Chronic pain affects many older adults, especially those with PD, and can be intense and frequent [158]. Acute pain resolves within months [159]; if it lasts three to six months or more, it is chronic pain [159,160]. Chronic pain can impair the quality of life for PwP [158,161,162]. PD pain is classified as musculoskeletal, dystonic, radicular/neuropathic, akathisia discomfort, and central pain. Musculoskeletal pain is the most common and is caused by rigidity and postural changes, often presenting as lower back pain, frozen shoulder, arthritis, or osteoporosis [162,163,164,165,166]. Chronic pain is also linked to depression, fatigue, sleepiness, and sleep disorders [167].

Managing chronic pain in PD is challenging [168,169], and patients often report dissatisfaction with their care [170,171,172]. It was suggested to use acetaminophen or other anti-inflammatories alongside carbidopa/levodopa or dopamine agonists for nociceptive pain [161], while opioids, carbidopa/levodopa, and gabapentinoids may be effective for central pain. Deep brain stimulation, rehabilitation, and alternative medicine can reduce pain, and methods such as exercise, massage, and acupuncture were also beneficial [169]. Furthermore, botulinum toxin and physical therapy can provide pain relief [166].

### 3.12. Current Overview of PD Treatment and Recent Advances

The current perspective on PD therapy extends beyond motor symptoms. Today, managing PD involves addressing both motor and non-motor symptoms through a proactive, wellness-focused approach [164,173,174,175,176]. The complexity of PD indicates that effective treatment in 2026 and beyond will likely rely on a targeted plan complemented by lifestyle modifications.

We have gained significant insight into the microenvironment underlying striatal dopamine function during PD progression. When reviewing the impact of levodopa treatment at different stages of PD progression, the challenge is to manage dopaminergic neuron loss with levodopa without the negative impact (levodopa-induced dyskinesia) often seen in advanced stages of PD [136]. Continuous therapy is needed to manage motor and nonmotor fluctuations [177]. Personalizing care is important to address the diverse motor and non-motor symptoms across the PD spectrum [178]. Gene therapy is a promising new approach that could eventually offer treatments that alter disease progression [175]. Finally, identifying and modifying lifestyle factors is an important strategy for preventing, modifying, and even treating PD [179].

Clinical Trials: Over the past five years, the latest developments in PD clinical trials have been reported [180]. They report that as of 31 January 2024, there were 136 active Phase 1–3 PD drug trials registered on ClinicalTrials.gov. Of these, 76 (56%) were classified as systematic treatment trials aimed at improving or reducing symptoms, while 60 (44%) were designated as disease-modifying treatments focused on delaying or slowing disease progression by targeting the underlying biology of PD [180]. More than half (58%) of the trials were in Phase 2, followed by Phase 1 (30%) and Phase 3 (12%). Many of these trials focus on dopaminergic agents, mainly targeting motor fluctuations such as off episodes with or without dyskinesia, levodopa-induced dyskinesia, cognitive function, depression, gait and balance issues, as well as psychosis or hallucinations [180].

Innovative Therapies: Nanoparticles are designed to enhance drug delivery across the blood–brain barrier, targeting PD-affected brain regions for more precise treatment [181]. Adaptive Deep Brain Stimulation uses an implanted device to monitor brain activity and deliver electrical pulses, adjusting responses based on symptom changes, with testing underway to improve symptom control and reduce medication use [182].

Wearable sensors for gait analysis in PwP have advanced, enabling assessments in natural environments to improve clinical evaluation [183]. New technologies are also emerging to identify abnormal gait patterns, potentially improving gait, balance, and mobility [183].

## 4. Neuropsychiatric and Cognitive Function

### 4.1. Anxiety

Anxiety is a common non-motor symptom of PD, not just a reaction. It manifests physically (sweating, headaches, sleep issues, racing heart, indigestion) and psychologically (panic attacks, worry, unease, nervousness, compulsive behaviors) [184,185,186]. Changes in anxiety to changes in hypothesized fear and limbic circuits were reported, suggesting PD neuropathology interacts with these pathways to promote anxiety.

Several prescription drug classes treat anxiety in PD (Table S4), but clear guidelines are lacking. These drugs have potential side effects, despite benefits for PwP with anxiety. SSRIs are most prescribed and have similar efficacy; sertraline is best tolerated and improves quality of life; SNRIs may be more effective but less well tolerated, though few studies assess SNRIs in PD [184]. Duloxetine aids chronic pain and FOG, while TCAs like nortriptyline and desipramine lower anxiety at lower doses than SSRIs, possibly reducing side effects.

### 4.2. Depression

Depression is common in PD, often involving apathy and anhedonia [187]. It may result from the impact on quality of life or the fear of PD outcomes. Besides the loss of dopaminergic neurons, noradrenergic and serotonergic neurons also decline [188], disrupting reward and mood regulation and possibly causing depression. It was suggested that depression in PD may stem from PD pathology, individual reactions, or both [189].

Antidepressant drugs are well-tolerated by individuals with PD [190,191]. Several medications help manage depression in PD (Table S4), emphasizing the importance of neurologists and psychotherapists in patient care. SSRIs or SNRIs should be first-line therapy. A Movement Disorder Review [191] suggested neurologists consider fluoxetine, sertraline, citalopram, venlafaxine, paroxetine, amitriptyline, nortriptyline, and desipramine for PD depression. Others recommend nortriptyline, venlafaxine, paroxetine, and citalopram, though results for paroxetine and citalopram are conflicting [192].

### 4.3. Apathy

Apathy is a lack of motivation and interest, and it is a relatively common non-motor symptom of PD [193]. Apathy is different from depression because it shows no sadness, although anxiety and depression can occur together in PD. A treatment plan for apathy in PD is to perform cognitive behavioral therapy to help develop coping mechanisms [193]. It is also vital to carefully regulate dopaminergic medication since apathy is thought to be related to losses of dopamine and serotonin.

### 4.4. Dementia and Psychosis

Many PwP will experience a decline in thinking and reasoning skills, which will be diagnosed as PD dementia (PDD) [194]. Impaired motor function usually appears first, followed by cognitive changes as α-syn deposits accumulate in the brain. Dementia typically occurs a year or more after these motor-related events. It is important to distinguish Lewy body dementia from PDD, which is characterized by motor dysfunction and the formation of Lewy bodies (α-syn deposits). Although medications can treat PDD (Table S5), starting and maintaining a regular exercise routine can help slow disease progression and delay the development of PDD [194].

PD psychosis (PDP) is a common neuropsychiatric condition where PwP experience hallucinations and delusions [195,196,197]. PDP is characterized by difficulties with attention, reality testing, language, and sensory perception [195]. Abnormal brain connections lead to false perceptions. Hallucinations in PD are linked to cortical atrophy in areas like the cuneus, precuneus, occipital gyrus, lingual and fusiform gyri, supramarginal and angular gyri, anterior cingulate, hippocampus, and thalamus [195]. Three antipsychotics treat PDP (Table S5).

### 4.5. Impulse Control Disorders

Impulse control disorders (ICDs) are a significant psychiatric complication of PD. They typically develop during dopamine replacement therapy, especially with dopamine agonists [134,198,199,200]. The most common manifestations of ICDs include compulsive gambling, compulsive shopping, hypersexuality, binge eating, and punding (repetitive, purposeless behaviors such as repetitive motor actions). If a PwP develops ICDs while on a dopamine agonist, the medication should be tapered off. Some PwP may resist discontinuing the dopamine agonist and may experience withdrawal symptoms after stopping the drug [201]. This is known as dopamine agonist withdrawal syndrome (DAWS) [201].

### 4.6. Impact of Stress on PD

Stress can worsen both motor and non-motor symptoms [202]. And dopaminergic medications are less effective for someone under stress. Mechanistically, stress releases dopamine from the basal ganglia. Thus, the key is to manage stress as best one can by exercising, practicing meditation, spending time with family and friends—anything that can relieve stress.

### 4.7. Cognitive Impairment

Cognitive impairment is a significant non-motor change in PD [203,204,205], which can progress to dementia [206]. It results from the loss of dopaminergic neurons in the substantia nigra and reduced dopamine in the frontal lobes. Cholinergic changes in the brainstem and corticostriatal pathways also contribute. The most common deficits involve attention, executive functions, memory, and visuospatial skills.

Three ways to improve cognitive deficits in PD are cognitive rehabilitation, lifestyle changes, and medication. Rehabilitation helps relearn or compensate. Before starting medication, people can adjust their exercise, diet, mental activities, sleep, and stress levels. Some beneficial drugs are Alzheimer’s medications like rivastigmine, donepezil, and galantamine (Table S5).

### 4.8. Managing Fatigue

Fatigue is a common, debilitating non-motor symptom in PD that reduces quality of life [207,208,209,210,211]. Fatigue can occur at any stage, even before motor symptoms. It is a subjective, persistent feeling of tiredness or low energy disproportionate to activity and not relieved by rest [207,211]. Fatigue is categorized as (i) physical, mental, and emotional; (ii) central (chronic, disease-related) and peripheral (muscle-related); and (iii) primary (isolated) and secondary (linked to mood or sleep) fatigue.

Fatigue management in PD uses tools such as the Fatigue Severity Scale and the Parkinson Fatigue Scale [212]. Exercise effectively combats fatigue [207,211]. Treatments include rasagiline [213], levodopa [214], and better sleep [215]. Nutritional supplements vary in benefit [216]. A personalized, multidisciplinary approach is recommended.

### 4.9. Executive Function Dysfunction

Executive function impairment is common in individuals with PD [203,217,218]. Executive functions are mental skills that support planning, organization, and independent behavior [219,220]. When executive functions are impaired, individuals may experience emotional instability, flat affect, impulsivity, irritability, poor personal hygiene, apathy (a lack of motivation), and difficulty with planning and goal-directed behavior. In normal aging, executive functions decline nonlinearly, with steeper declines after age 60 [221]. Notably, reductions in attention and increases in executive function can contribute to motor problems that may lead to falls [222].

PwP with executive function issues could struggle to plan their day, get ready for work, start new activities, follow a recipe, read a map, or find the right words during conversation. The prefrontal cortex supports executive functions and is influenced by dopaminergic, noradrenergic, serotonergic, and cholinergic inputs [223].

Executive dysfunction involves multiple brain regions, and proper functioning requires access to various neurotransmitters, including those in the dopaminergic system. Dopaminergic therapy should be carefully considered for PD patients [224]. Daily activities such as reading aloud, cognitive games, exercise, mindfulness, creative pursuits, social interactions, and planning can support executive functions. Chess is particularly beneficial because it engages brain regions involved in recognition and spatial awareness.

### 4.10. Current Overview of PD Neuropsychiatric and Cognitive Function and Recent Advances

The current perspective on PD emphasizes identifying and managing its neuropsychiatric components [225,226,227]. Although motor symptoms often dominate the conversation about PD, non-motor issues such as anxiety, depression, apathy, psychosis, and cognitive decline significantly impact quality of life [225,228]. Therefore, it is essential to thoroughly evaluate each patient to assess these neuropsychiatric and cognitive features.

Neuropsychiatric and Cognitive Function Studies: The link between cognitive functions and dysautonomia was studied in de novo PD, using MoCA-K (Korean version) and Seoul Neuropsychological Screening for cognition, and SCOPA-AUT for autonomic functions [229]. They found a strong connection between autonomic dysfunction (e.g., gastrointestinal issues), and cognitive impairment. Monitoring dysautonomia in early PD can help identify patients at risk of cognitive decline.

Anxiety, depression, and sleep issues are common in PD [230]. PD patients reported more depression, especially with sleep problems, which also worsened anxiety. Factors like age, motor symptoms, and sleep disturbances relate to depression, while sleep issues are linked to anxiety. Overall, depression, anxiety, and sleep disturbances frequently occur together in PD.

## 5. Sleep Challenges

### 5.1. Insomnia

Insomnia is difficulty falling and staying asleep, often worsened by early awakenings, leading to fatigue, low motivation, and irritability [231]. It is the most common sleep disorder in PD, increasing with disease progression [232]. Insomnia links to lower quality of life, higher levodopa doses, cognitive decline, and depression [233].

Treatment options include sleep hygiene, cognitive behavioral therapy, aiming to achieve continuous dopaminergic delivery to improve PD symptoms, and carefully evaluating coexisting conditions such as chronic pain, depression, anxiety, and restless leg syndrome [231,232]. Melatonin helps treat insomnia in PD [234].

### 5.2. Excessive Daytime Sleepiness

A PwP with excessive daytime sleepiness exhibits persistent daytime sleepiness, leading to brief sleep episodes [235,236]. The PwP anticipates this sleep but cannot control it. Some of these sudden events are referred to as ‘sleep attacks,’ and they occur without warning of drowsiness [237]. These patients need more levodopa, often have sleep apnea, and show higher rates of depression, hallucinations, and dementia [231].

Managing excessive daytime sleepiness involves evaluating medications like levodopa, dopamine agonists, antidepressants, insomnia drugs, and antipsychotics that can cause drowsiness. Diagnosis includes taking a history, performing sleep tests, identifying sleep-inducing medications, managing comorbidities such as sleep apnea, reviewing all medications, and possibly using wake-promoting drugs such as modafinil or solriamfetol [238].

### 5.3. Circadian Dysfunction

Circadian rhythms are innate timekeepers that regulate physiological processes at optimal times [239], that are controlled by the biological clock in the suprachiasmatic nucleus (SCN). Disruptions in PD are increasingly significant in non-motor sleep disorder research [240]. PwP often experience disrupted sleep–wake cycles, with earlier sleep and wake times than desired [232,238].

PwP with circadian dysfunction are usually in advanced disease stages, often with depression and dementia. Causes involve degeneration of retinal dopaminergic cells, the SCN, and the pineal gland, disrupting circadian rhythms. Treatment involves managing sleep–wake schedules, controlling light exposure, and using external melatonin supplements. In some cases, medications such as lorazepam may be appropriate. As our understanding of circadian disruption in PD advances, our capacity to treat this disorder will improve [231,239].

### 5.4. Restless Legs Syndrome (RLS)

According to the International Classification of Sleep Disorders (ICSD-3), Restless Legs Syndrome (RLS) is a sleep-related movement disorder characterized by an urge to move the legs that worsens with inactivity and improves with movement [231,241,242,243]. RLS in people with PwP resembles that in those without PD. RLS and PD are connected through dopamine dysfunction, and both conditions respond to dopaminergic therapy [244]. Due to the link between iron deficiency and RLS, serum iron levels should be checked regardless of PD status [245].

Improving sleep hygiene is the first step, along with addressing iron deficiency and any other factors that may contribute to RLS. Another option would be increasing dopaminergic medication, typically a dopamine agonist [231].

### 5.5. Obstructive Sleep Apnea (OSA)

OSA is a repeated obstruction of the upper airway during sleep, where breathing is blocked in the throat, leading to sleep disruption [246]. It is commonly associated with obesity, snoring, nocturia, and excessive daytime sleepiness. However, in PwP, obesity is rarely present despite OSA [247].

The high prevalence of OSA may result from aging, extended sleep duration in the supine position (lying on your back), and increased rigidity of the oropharyngeal muscles. OSA in PD might be more linked to aging than directly caused by the disease itself.

OSA is usually diagnosed through video polysomnography. The standard treatment for OSA involves weight loss if needed, mandibular advancement therapy, and continuous positive airway pressure (CPAP) therapy.

### 5.6. Rapid Eye Movement (REM) Sleep Behavior Disorder (RBD)

RBD is characterized by nightmares involving threats or chases, with dream-enacting movements like jerking, thrashing, or shouting [231,246]. It results from dysfunction of the subcoeruleus and magnocellularis nuclei in the brainstem [248,249,250], which normally inhibit muscle activity during REM sleep, thereby causing atonia.

Diagnosing RBD in PD requires video polysomnography to detect REM activity linked to dream behaviors [251], crucial for distinguishing it from other disorders like OSA. RBD can be an early PD sign, with severe symptoms like older age, gait freezing, orthostatic hypotension, sleepiness, impulse issues, hallucinations, and dementia [116,252,253,254].

Managing RBD in PD begins by securing the bed environment to reduce the risk of injury to both the patient and the bed partner. Avoid medications that worsen RBD symptoms, such as antidepressants and beta-blockers. While dopaminergic drugs generally do not affect RBD, safinamide may be beneficial, likely due to its anti-glutamatergic properties. For PwP with RBD, acetylcholinesterase inhibitors like donepezil and transdermal rivastigmine can be helpful [255].

### 5.7. Current Overview of PD Sleep Disorders and Recent Advances

Current estimates suggest that ~90% of PwP are affected by sleep disorders [256]. Thus, sleep disorders are a very important non-motor symptom of PD and deserve a treatment strategy for each patient, especially since poor sleep quality accelerates cognitive decline and generally worsens other symptoms (e.g., tremor) [256,257]. The glymphatic system transports cerebrospinal fluid and removes excess interstitial fluid and proteins; its activity is significantly increased during sleep [258]. Growing evidence suggests that the glymphatic system functions poorly in PD [259].

It is important to note that certain sleep disorders may serve as early warning signs of PD. RBD can precede the clinical manifestation by one or even two decades and is considered a highly predictive prodromal marker of synucleinopathies [260,261]. RLS may also precede motor onset by several years, although with lower predictive strength than RBD [262,263]. Therefore, early recognition of these alterations contributes to the prodromal assessment of PD and other synucleinopathies.

Sleep Studies: Recently, it was asked whether OSA was associated with an increased risk of incident PD diagnosis among US veterans [264]. They also examined whether continuous positive airway pressure (CPAP), the standard treatment for OSA, affected PD risk. Their results showed that OSA was an independent risk factor for later PD and that early CPAP treatment could modify this risk [264].

Changes in and interactions between subjective sleep and cognition following deep-brain stimulation (DBS) were studied in PD [265]. Their results showed that DBS was highly beneficial for improving sleep and reducing sleepiness [265]. However, they found no evidence that DBS altered cognition.

## 6. Exercise and Movement

### 6.1. Impact of Exercise on Healthy Aging and for Those with Neurodegenerative Diseases

Exercise can help reduce the effects of age-related decline [266,267,268,269]. Although aging cannot be reversed, exercise supports or improves several key physiological functions, including cardiovascular and brain health, bone density and muscle mass, mental health, sleep quality, and overall quality of life.

The Active People, Healthy Nation™ recommendation applies to adults aged 18–64 and those 65 and older, suggesting 150–300 min of moderate-intensity or 75–150 min of high-intensity exercise per week, plus muscle-strengthening activities twice a week [270].

Regular exercise can improve symptoms of neurodegenerative disorders like PD and Alzheimer’s disease [271,272,273,274,275,276,277,278,279,280,281]. It benefits brain health, cognition, and quality of life, while inactivity has adverse effects [282,283,284,285].

We recently reviewed the benefits of exercise for PD, covering aerobic exercise, resistance training, neuromotor exercises, and stretching and flexibility exercises to improve motor and non-motor symptoms, gait and posture, and reduce fall risk (see Ref. [286] and work cited therein).

### 6.2. Neurobiological and Molecular Foundations of the Benefits of Exercise in PD

In recent years, exercise has emerged as a highly effective non-pharmacological strategy for managing PD. Movement affects the nervous system by restructuring neural circuits, altering molecular pathways, and directly influencing biological processes linked to disease development [283,284,287,288,289,290]. Recognizing these mechanisms allows us to view exercise not only as a supplementary treatment but also as a vital part of therapy.

Neuroplasticity: Exercise triggers neurotrophic factors that support neuronal survival and growth. The most well-known is BDNF (Brain-Derived Neurotrophic Factor), which boosts synaptic plasticity, helps form new neural connections, and strengthens the resilience of dopaminergic neurons—especially vulnerable in PD [288]. In addition, GDNF (Glial Cell Line–Derived Neurotrophic Factor) provides targeted protection within the substantia nigra [289], while VEGF (Vascular Endothelial Growth Factor) enhances cerebral blood vessel growth [291]. Essentially, exercise fosters a biological environment that favors neuronal health [283].

Effects on the dopaminergic system: Recent research indicates that exercise can directly influence basal ganglia physiology [290,292,293]. Engaging in physical activity increases the expression of tyrosine hydroxylase, a key enzyme in dopamine production, thereby enhancing neurotransmitter regulation [294]. Other research shows that exercise restores D2-like dopamine receptors [295]. Exercise restored dopamine transporter (DAT) levels in the substantia nigra and putamen, and increased neuromelanin levels within the substantia nigra [296]. Collectively, these studies imply that high-intensity exercise has a “restoration” effect on the dopaminergic system [289].

Reduction in oxidative stress and neuroinflammation: PD features a pro-oxidative and pro-inflammatory environment in the brain [297]. Exercise helps combat these processes by boosting the body’s own antioxidant defenses, lowering free radical production, and reducing microglial activation [298,299]. This, in turn, diminishes the release of inflammatory cytokines [300]. The systemic and central anti-inflammatory effects support the slowing of degenerative changes and enhance neuronal function [301].

Improvement of mitochondrial function: Dopaminergic neurons are particularly susceptible to energy deficits. Exercise stimulates mitochondrial biogenesis via the PGC-1α pathway, enhances respiratory chain efficiency, and reduces apoptosis [302,303,304]. In short, exercise boosts the neuron’s “power plant,” making it more resilient to metabolic stress.

Modulation of glutamate and motor circuits: In PD, high levels of glutamatergic activity contribute to symptoms like rigidity, bradykinesia, and freezing of gait. Exercise helps reestablish the balance between excitatory and inhibitory signals in the basal ganglia, decreasing excitotoxicity and enhancing movement [305,306,307,308]. This explains why activities such as Rock Steady Boxing, Tai Chi, PWR!Moves, dancing, brisk walking, or cycling have evident benefits for motor fluidity.

Systemic effects that influence the brain: Beyond strictly neuronal mechanisms, exercise provides systemic benefits that positively affect brain health [305,306,307,308]:

- exercise improves cerebral perfusion and oxygenation,
- enhances the glymphatic system, facilitating the clearance of toxic proteins,
- regulates sleep, amplifying nighttime metabolic waste removal,
- reduces cortisol and chronic stress, and
- improves immune system function.

Recommendation: Exercise should be regarded as more than a suggested activity for PwP. Current evidence demonstrates that exercise is a biologically active intervention capable of altering molecular processes involved in PD. Exercise has been shown to influence neuronal plasticity, dopaminergic activity, mitochondrial function, neuroinflammation, and motor circuits. In addition, exercise can improve both quality of life and clinical stability for PwP. Overall, exercise is a therapeutic modality that complements and enhances the benefits of pharmacological and rehabilitative treatments for PD.

### 6.3. Seek Expert Guidance Before Starting an Exercise Routine

**Figure 5:**
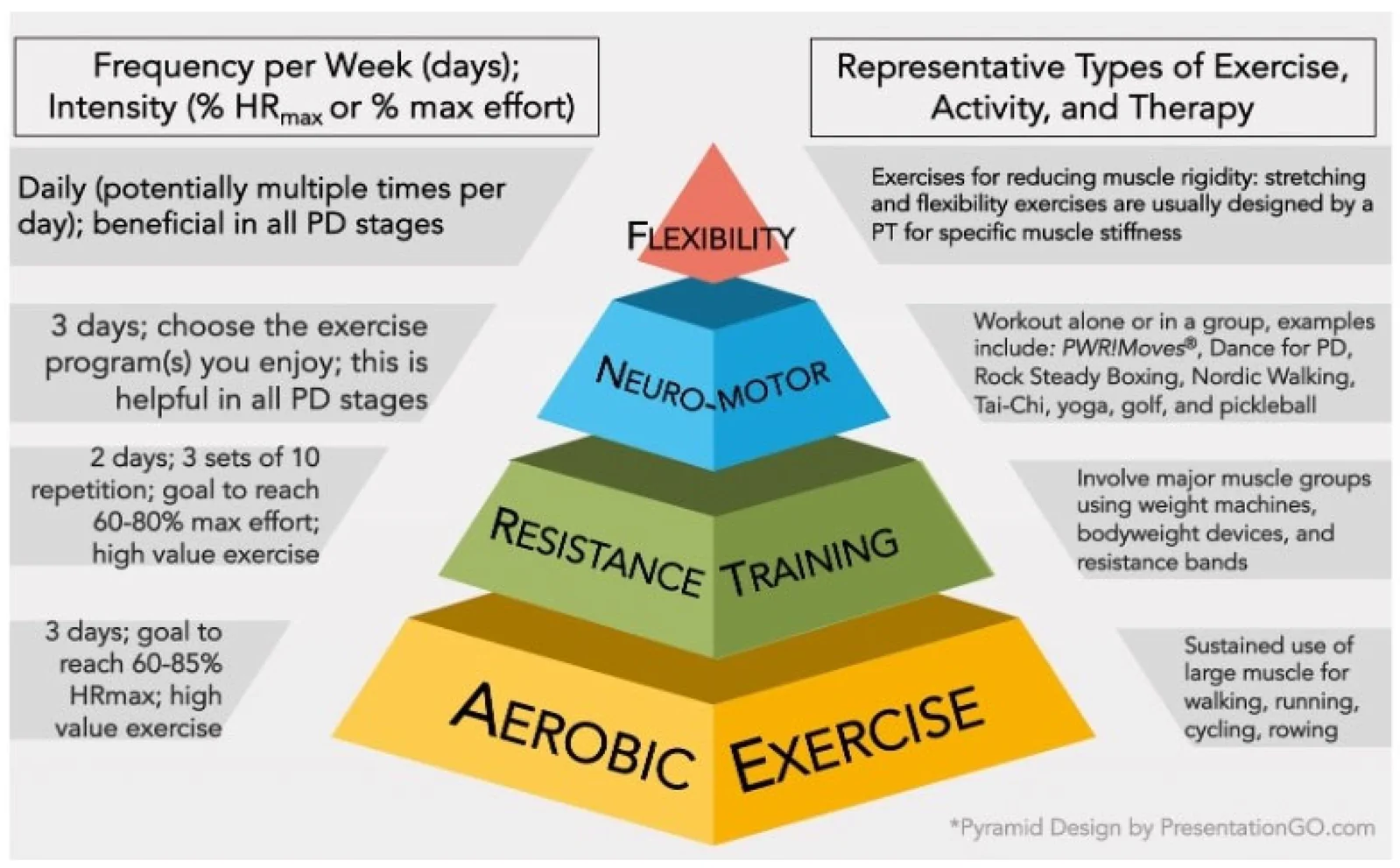
The PD exercise pyramid (*pyramid design by PresentationGo.com) includes four types of exercise: aerobic exercise, resistance training, neuromotor therapy, and stretching/flexibility exercises. On the (**left**) side are the recommended schedules, and on the (**right**) side are the exercises best represented by each category. This figure was used with permission [286].

Exercise can help manage PD at any age by improving mobility and mental health and possibly slowing disease progression. Consult a doctor or a physical therapist before starting, especially if new to exercise or having conditions like heart disease, diabetes, or kidney disease. Regular exercise is more beneficial than either occasional exercise or sedentary activity. A tailored program can improve symptoms and management. Plans should match each person’s needs, interests, and PD stage. Figure 5 illustrates the main types of exercises suggested for PwP.

### 6.4. Current Overview of PD Exercise and Movement and Recent Advances

The up-to-date view of exercise and movement continues to underscore its importance for individuals with PD in maintaining quality of life and even slowing its progression. Importantly, two major groups jointly published updated exercise guidelines. The American College of Sports Medicine (ACSM) [309] and the Parkinson’s Foundation [310] mandate a minimum of 2.5 h (150 min) of exercise per week, divided into four types of exercise. Evidence is mounting that regular, vigorous exercise can slow disease progression, provide a template that promotes neuroplasticity, and enhance dopamine signaling.

Exercise Studies—Intensive exercise induced a state of neuroplasticity in response to neurotoxic α-syn polymers, and exercise reduced the rate of disease progression, which is slowly evolving due to the pathologic α-syn aggregates [294].

Most exercise programs emphasize motor skills, often neglecting non-motor symptoms (NMS). Recent research examined the association between self-reported exercise and NMS, including depression, anxiety, cognition, fatigue, and daily non-motor experiences [311]. The study found that individuals who reported exercising had fewer and milder NMS than those who did not exercise [311].

Tai Chi is a mind−body practice with slow movements, weight shifting, and proper posture. Tai Chi was found to be effective, alongside resistance and stretching, for improving motor symptoms, physical abilities, and quality of life in early to mid-stage PD [312]. Furthermore, Tai Chi especially benefits muscular endurance, motor control, and balance.

## 7. Striving for Wellness

### 7.1. Health and Wellness

The World Health Organization (WHO) defines health as “a state of complete physical, mental, and social well-being, and not merely the absence of disease or infirmity. It is a resource for daily life, enabling people to manage stress, work effectively, and contribute to their community.” [313]. The National Institute of Wellness defines wellness as “an active process through which people become aware of, and make choices toward, a more successful existence.” [314,315]. Applying these definitions to living well with PD [316,317,318,319], we see health as a state of being, with wellness in PD involving active lifestyle choices that promote health.

### 7.2. Skillful Living

‘Striving for normality’ in PD involves maintaining independence and focusing on the present [320]. Living with PD also means avoiding situations that could worsen the disease [321,322,323,324].

An individual’s quality of life depends in part on their ability to live well with PD [316,325,326]. Wellness can be pursued through medical, social, psychological, and lifestyle approaches [327]. Planning each day to maximize productivity when medication works is crucial. If PD manifests unexpectedly, adjust routines to manage episodes. Resilience and flexibility are vital, as they enable recovery and better well-being.

### 7.3. Diet and Dietary Inflammatory Index

A balanced diet rich in fruits, vegetables, and whole grains can improve well-being and help manage symptoms [328]. Fresh produce, nuts, seeds, non-fried fish, olive oil, wine, coconut oil, herbs, and spices were associated with slower PD progression, whereas canned foods, soda, fried foods, beef, ice cream, yogurt, and cheese were associated with faster PD progression [328].

Inflammation may contribute to aging and brain disorders [329], and diet is a key preventive measure [330]. However, diet can also promote inflammation, as measured by the Diet Inflammatory Index (DII) [331]. The DII correlates with markers such as IL-1β, IL-4, IL-6, IL-10, TNF-α, and CRP, which may influence the risk of neurodegenerative diseases like PD [329,330,331].

### 7.4. Complementary and Alternative Medicine (CAM)

Many PwP use CAM as adjunctive therapy for PD. The National Institutes of Health defines CAM as medical products and practices not part of standard care. ‘Complementary medicine’ is used alongside standard treatment, whereas ‘alternative medicine’ replaces it.

Although providing an in-depth overview of CAM in PD is beyond the scope of this review, the five categories that form the principles of CAM are presented:

Alternative medical systems include traditional Chinese medicine, Ayurveda, and naturopathic medicine.

Mind–body interventions include mindfulness meditation.

Biologically based therapies include many over-the-counter (OTC) products, nutraceuticals, and herbal therapies.

Manipulative and body-based therapies include Reiki and therapeutic touch.

Energy therapies include chiropractic and massage therapies.

An example of CAM was the use of ultra-micronized palmitoylethanolamide, which reduces neuroinflammation, offers neuroprotection, and slows PD progression and disability in advanced patients [332]. See references [333,334,335,336,337,338,339,340] for more information on CAM.

### 7.5. Personality Traits and Attitude

Evidence suggests that certain personality traits and attitudes can promote normalcy and wellness in the presence of PD.

Resilience: Resilience mitigates the effects of stress and promotes adaptation. Resilience protects against disability and supports quality of life in PwP [341]. Enhancing resilience could be a beneficial treatment strategy. Resilient PwP have more active lifestyles, increased gray matter in motor regions, stronger connectivity, and maintain motor function for a decade [342].

Hope: Hope was significantly correlated with health-promoting actions, spiritual growth, and interpersonal support [343]. PwP value social identities and activities for well-being and hope [344].

Gratitude: Participants who focused on gratitude consistently experienced more positive emotions and greater life satisfaction than those who focused on daily annoyances or neutral events [345]. In other words, being thankful can improve health all on its own.

Optimism: Maintaining optimism and positive self-perception can affect future outcomes [346]. They recommended helping patients cultivate optimism and hopefulness to foster positive views of well-being. Similarly, acceptance of the diagnosis is crucial for PwP to foster optimism [319].

Resilience, hope, gratitude, and optimism are fundamental to positive psychology. They serve as both protective traits and learnable skills that boost well-being and help individuals manage life’s difficulties [347]. It is important to maintain a positive outlook despite the challenges posed by a severe disease such as PD, while proceeding with caution [348]. Thus, it is crucial to educate newly diagnosed PwP about these protective traits, as they can provide encouragement on their wellness journey.

### 7.6. Wellness Strategy for PD

Like PD, each person’s wellness plan varies based on age, health outside PD, life stage, disease progression, and acceptance of PD. However, core principles can guide PD wellness strategies: regular exercise, sleep routines, balanced diet, collaboration with healthcare providers, and mental health support when needed. This approach aims to optimize brain health (potential for neuroplasticity), manage symptoms (and potentially slow progression), and improve overall wellness, ultimately enhancing quality of life for those with PD.

### 7.7. Current Overview of PD Wellness and Recent Advances

The current view is that the best path to PD wellness is a focused, proactive, and more holistic approach [349]. Significant exercise can slow PD progression, a benefit beyond the capability of any known substance or medical treatment [350]. Furthermore, it is now important to follow a proper diet focused on improving gut–brain health [351]. Given the high prevalence of depression, anxiety, and apathy in PD, the goal is to seek tools to manage these aspects of PD. Additionally, stay connected with people through community support and other means to counteract the social isolation that can occur over time with PD [350].

Wellness Study: A study measured motor and non-motor symptom changes in PD participants after the 8-week ‘PWR!Moves’ program, a multidisciplinary wellness course [352]. Most improved in three of four UPDRS areas. Results support holistic programs to boost occupational participation and highlight the need for PD interventions that evaluate motor skills, psychosocial health, and social ties [352].

Preventive Neurology: A lifelong exposure to pollutants, chemicals like pesticides, stressful lifestyles, and dietary exposure factors likely contributes to increased PD [353]. Some have suggested shifting toward preventive brain health [354,355]. The American Academy of Neurology’s Brain Health Initiative is training neurologists in prevention [356]. Five steps to promote preventive brain health, inspired by successes in cardiovascular disease prevention, were recently described [357].

## 8. Obstacles/Paths Forward

### 8.1. Does PD Describe a Single Disease or a Spectrum of Biological Subtypes?

The current clinical classification is: Idiopathic PD (the most common, with an unknown cause), Familial PD (genetically inherited), and Atypical Parkinsonisms (“Parkinson-plus” syndromes). Given the large number of motor and non-motor symptoms, PD would benefit from a subtype classification system [358]. While creating such a subtype list for PD is daunting, one could envision future success in treating and understanding the disease, especially as it relates to therapy and prognosis. The current classification state of PD resembles the early phase of breast cancer, when ‘curing’ the disease was extremely challenging before the identification of specific subtypes, such as ER/PR-positive, HER2-positive, and triple-negative breast cancer, which eventually led to targeted therapy.

Our existing drugs cannot stop PD from being a chronic, progressive condition. Thus, it is essential for healthcare professionals currently managing PwP to stay informed about potential changes in category definitions. The development of PD subgroups is hoped to enable targeted therapies and improve quality of life.

### 8.2. Why Have Disease-Modifying Drugs Failed So Far?

Calling them failures may be too harsh a way to describe their ineffectiveness. However, one could say that, therapeutically, the target was good, but the drug’s aim was off [359]. The vast heterogeneity of PD, as mentioned earlier, has likely hampered many clinical trials [360]. Furthermore, the blood–brain barrier is designed to protect the brain by preventing many substances from entering it. Thus, navigating the barrier and successfully treating the neurological disease are difficult tasks. Other reasons include that many of these studies have been based on rodent-derived data, but these models do not naturally develop PD, and when PD is induced, its progression differs from that in humans. However, if PD subtypes can be effectively created, one prediction is that further disease-modifying drugs will begin working, and advances in treating PD will occur.

A recent phase 1b open-label study examined NT-0796, an oral drug that inhibits the NLRP3 inflammasome, in subjects with PD [361]. NT-0796 reduces CNS inflammation by targeting neuroinflammation driven by aggregated α-syn, which promotes the production of pro-inflammatory cytokines such as IL-1β in microglial cells, thereby contributing to dopaminergic cell death. Results from 10 PwP and 4 controls showed NT-0796 lowered inflammation over 28 days, paving the way for longer studies on PD neurodegeneration [361].

### 8.3. What Is the Relevance of the Microbiota–Gut–Brain Axis for PD?

The gut–brain axis is a complex, two-way communication system involving biochemical and cellular signals that connect the central nervous system (CNS) with the enteric nervous system (ENS), which consists of neurons in the walls of the digestive tract that regulate digestion [362,363]. Recent research has shifted from just observing correlations to uncovering the underlying molecular and causal mechanisms [364]. It was traditionally thought that the gut mainly communicated with the brain through hormones and cytokines slowly released into the bloodstream. However, the discovery and detailed mapping of neuropod cells in the intestinal lining revolutionized this understanding [365,366,367]. These sensory cells form direct synapses with the vagus nerve and use neurotransmitters like glutamate and serotonin to rapidly send signals about nutrients and toxins in the gut to the brain within milliseconds—matching the speed of brain synapses [365,366].

The gut microbiota ferments dietary fiber to produce key metabolites like acetate, propionate, and butyrate—short-chain fatty acids (SCFAs) [368]. Recent research indicates that SCFAs serve as powerful epigenetic regulators and help maintain the integrity of the blood–brain barrier (BBB). When intestinal microbiota becomes imbalanced (dysbiosis), butyrate levels decrease, causing a ‘leaky gut.’ This increased permeability allows bacterial lipopolysaccharides (LPS) and pro-inflammatory cytokines to enter the bloodstream, cross the BBB, and activate microglia, ultimately leading to chronic neuroinflammation that may foster cognitive decline.

Recent pathological findings closely align with Braak’s hypothesis on the trans-synaptic spread of α-syn [369,370]. α-Syn appears to misfold initially within the ENS, often triggered by toxins or local bacterial infections. This toxic protein behaves like a prion, traveling retrograde along vagus nerve fibers to the dorsal motor nucleus in the brainstem, then spreading to the substantia nigra. Current clinical approaches suggest that blocking this pathway early could prevent progression of motor symptoms.

Gut bacteria act as living chemical bioreactors [371]. Specific species within the *Lactobacillus* and *Bifidobacterium* genera autonomously synthesize GABA and acetylcholine, while the *Candida*, *Streptococcus*, and *Escherichia* genera produce serotonin (in fact, about 90% of the body’s total serotonin is manufactured in the gut) [372].

In 2026, psychiatry and neurology are validating the first precision psychobiotics (engineered or selected bacterial strains) as adjuvant treatments for major depressive disorder, autism spectrum disorder (ASD), and generalized anxiety disorder, directly modulating brain receptors through microbial metabolism [371,373].

### 8.4. How Can We Better Use Paradoxical Kinesia to Improve the Quality of Life for PwP?

There are several interesting aspects to the paradoxical kinesia phenomenon [374,375], including (i) the wheelchair-bound patient immediately able to walk or run when in a life-threatening scenario; and (ii) a patient with advanced disease barely able to walk but can effortlessly pedal a bicycle. Clearly, the first example is driven by adrenaline to simulate motion, and the other is the brain adapting and finding new motor pathways. Regardless, the process has bypassed the PD-driven and damaged dopaminergic neurons in the basal ganglia, allowing a PwP to regain motor function.

Physical therapists have used various techniques (e.g., cue-based movement, visual targets, and rhythmic cues) to help retrain patients with frequent freezing episodes and allow them to walk again [376]. Furthermore, walking while concentrating on bouncing a lacrosse ball or rotating a soccer ball around your waist can often correct a patient’s walking pattern to resemble a more normal gait (F.C.C., unpublished observation). This suggests that the PD brain may be fooled into believing the gait is fine, although this is only a temporary adjustment. It suggests that therapists should continue to focus on improving these gait complications and make the exercises/techniques/devices available to everyone.

### 8.5. What Is the Role of Protein Aggregation in the Pathophysiology of PD?

There are many proposed mechanisms underlying the pathological process of PD, including environmental toxins [110], genetic [109], immunologic [377], neuronal vulnerability [378], mitochondrial-driven [107,379], lysosomal-driven [380], neuroinflammation [108], lipidopathy [381], proteinopathy [381], neural circuit dysfunction [382], oxidative stress [108], prion-like [106], and α-synucleinopathy [383,384].

Many of these proposed mechanisms can be unified under what we call the α-syn pathogenic process. This does not negate other pathways but underscores α-syn’s role in the broader pathophysiology of PD. Targeting α-syn aggregation, neuroinflammation, oxidative stress, or immune responses may slow PD progression; however, effective management likely requires addressing multiple pathways simultaneously. This multicomponent driver of PD pathophysiology highlights the difficulty of slowing progression, let alone curing the disorder.

**Figure 6:**
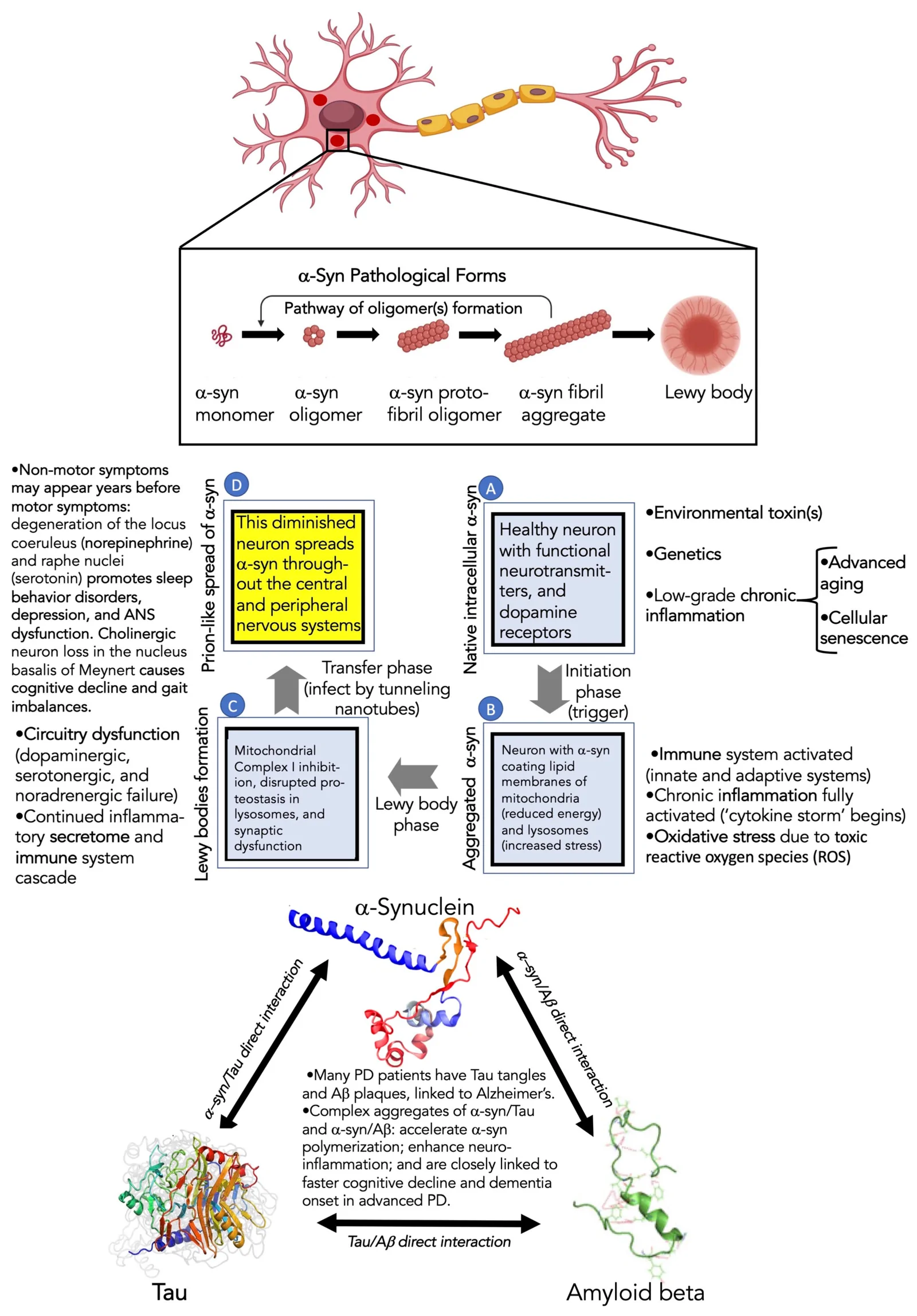
The α-synuclein (α-syn) pathogenic process in PD. The (**top panel**) depicts the structural pathological progression of α-syn aggregation. This part of the figure was modeled after [389]. The (**middle panel**) shows three phases: (**A**) to (**B**) Initiation—environmental toxins, genetic defects, aging, and cellular senescence trigger α-syn changes, leading to organelle membrane coating, energy loss, and stress; (**B**) to (**C**) Lewy body formation—α-syn aggregates cause immune activation, inflammation, ROS production, and mitochondrial and lysosomal dysfunction; (**C**) to (**D**) Transfer—Lewy bodies form, depleting nutrients and lipids, inhibiting ATP production, disrupting autophagy, and causing apoptosis. α-Syn transfers through tunneling nanotubes, spreading pathology. The (**bottom panel**) describes the proteinopathy pathway in which α-syn forms aggregates with Tau and Aβ, thereby promoting additional aggregation within a PD neuron. Structural drawings were based on the following: α-syn [390], Aβ [391], and Tau [392].

The α-syn pathogenic process was synthesized from several reviews [73,359,384,385,386,387,388,389]. α-Syn has distinct roles at various stages of PD, all of which are influenced by structural changes in the protein (Figure 6). Thus, α-syn provides the PD process with leverage (or control) as it progresses from monomer to aggregate to Lewy body. Although α-syn might initially protect the neuron, it eventually accelerates disease progression by forming insoluble aggregates that harm neurons and cause cell death (Figure 6).

The aggregation of α-syn into Lewy bodies, characteristic of synucleopathies, also occurs in dementia with Lewy bodies (DLB) and multiple system atrophy (MSA) [60]. Both DLB and MSA are chronic, progressive neurodegenerative diseases. Several other neurodegenerative diseases share similar protein-aggregation mechanisms, but different proteins mediate this process across various brain regions [393]. Alzheimer’s disease (AD) involves amyloid-β plaques outside of neurons and tau tangles inside [394]. Amyotrophic lateral sclerosis (ALS) involves misfolded TDP-43 and sometimes FUS or SOD1 [394]. Frontotemporal lobar degeneration features tau or TDP-43 aggregation. Huntington’s disease results from clumping of the mutant huntingtin protein. Prion diseases (e.g., Creutzfeldt-Jakob Disease) are caused by infectious misfolded prion proteins.

Recent neurobiochemical studies have detected multiple proteinopathies in PD (Figure 6) [395]. Although amyloid-beta (Aβ) and Tau protein are historically considered markers of AD z, they have also been shown to contribute to the pathogenesis of PD [396]. The interaction between Tau and Aβ with α-syn synergizes to accelerate network collapse and promote the transition to cognitive decline, a phenomenon especially relevant in PDD [397]. High levels of phosphorylated Tau (p-Tau) in the cerebrospinal fluid (CSF) and polymorphisms in the MAPT gene (which encodes Tau) are strongly associated with faster motor decline and early-onset cognitive deficits in PD. Amyloid acts as a “trigger” such that deposits in the cortex create a neurotoxic microenvironment that accelerates the propagation of Lewy pathology (α-syn) from subcortical structures to the neocortex [397]. Amyloid co-pathology is the most powerful biological predictor of dementia in PD. Low CSF Aβ levels directly correlate with the development of cognitive and visuospatial impairments in PD. Furthermore, growing evidence suggests that misfolded TDP-43 (found in ALS) can also interact with α-syn [398,399].

Currently, advanced PD or PDD is interpreted as a triple proteinopathy involving α-syn, Tau, and Aβ [397,400,401]. The triadic interaction among these proteins follows a precise model: α-syn initiates synaptic damage and subcortical motor neurodegeneration; cortical Aβ facilitates the anatomical progression of alpha-synuclein toward cognitive areas. The Tau protein, synergizing with both, amplifies axonal damage and induces definitive neuronal death in both the substantia nigra and the cortex. The cross-over of Tau, Ab, and TDP-43 with α-syn into the pathology of PD adds to the complexity of PD; however, it also underscores the continued importance of developing sensitive, accessible biomarker assays to characterize these deficits in α-syn-driven PD.

### 8.6. How Close Are We to Developing Effective Biomarkers for PD?

Consider these diseases and their specific biomarkers: type 2 diabetes is monitored using hemoglobin A1c; prostate cancer is assessed using prostate-specific antigen (PSA); and some cardiovascular diseases are measured using low-density lipoprotein cholesterol (LDL-C) and high-density lipoprotein cholesterol (HDL-C) levels. While these tests are not perfect, their routine use has helped many patients monitor and treat their respective diseases.

Diagnosing PD can be difficult, and useful biomarkers could improve many aspects of managing PwP. Imaging and genetic tests can assist in diagnosing PD; however, no reliable biological test is currently available. Recently, a new test has been developed to measure very low levels of α-syn, called seed amplification assays (SAAs) [402]. The SAA test measures α-syn’s ability to induce self-aggregation in cerebrospinal fluid (CSF). Other targets (e.g., a marker for mitochondrial DNA damage and the microRNA miR-44438) and new imaging biomarkers and screening methods are currently being developed [402]. The possibility that SAAs can distinguish prodromal and preclinical disease stages is exciting.

### 8.7. Solving the Puzzle of PD

**Figure 7:**
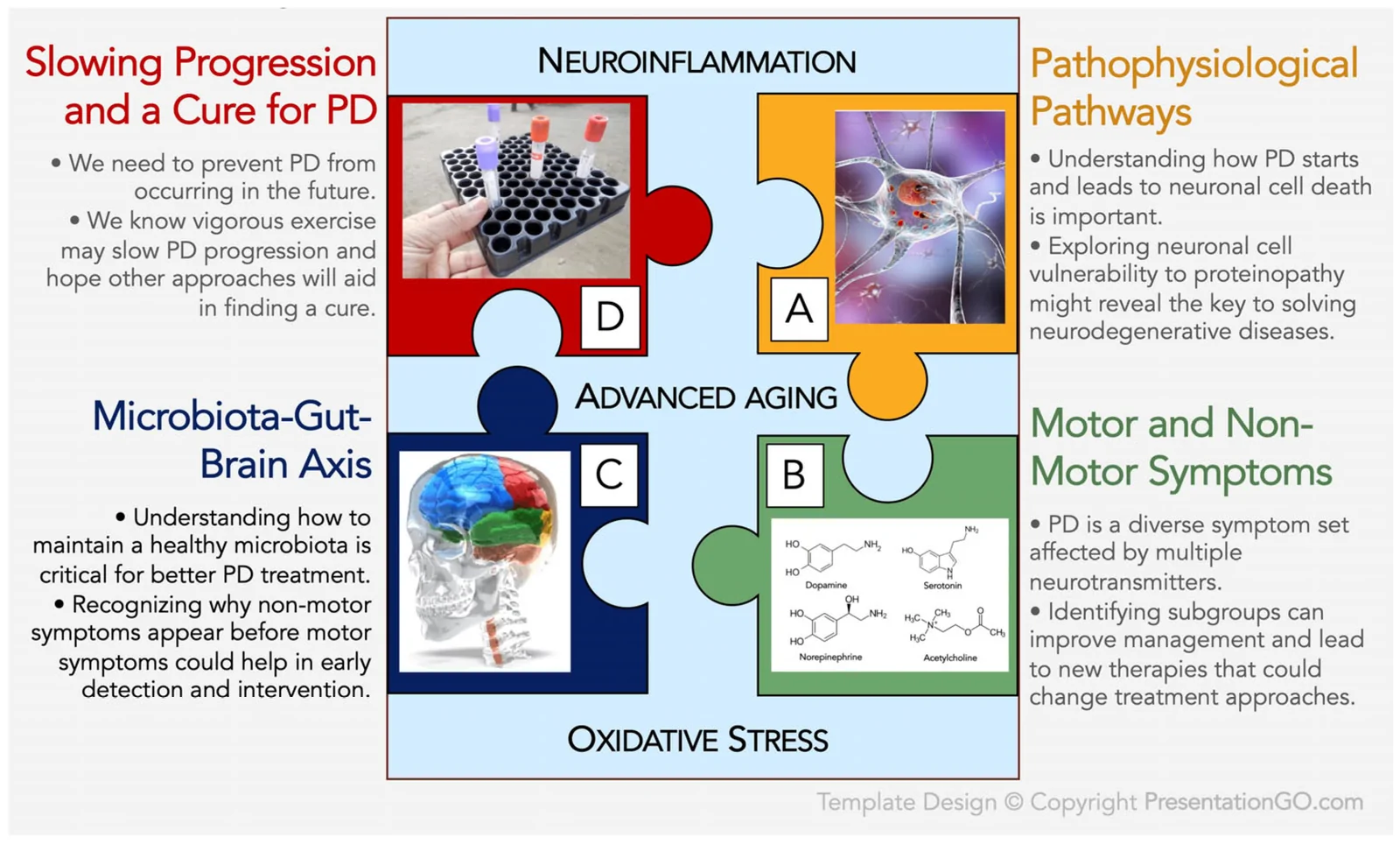
Examples of obstacles and potential paths forward for solving the complex puzzle of PD. (**A**–**D**) show some of the categories that need to be addressed, under the constant backdrop of the detrimental roles played by neuroinflammation, advanced aging, and oxidative stress.

We have learned much about the basic science and clinical aspects of PD. We know today that PD is not simply a dopamine deficiency; PD is now thought to be a multiorgan and multisystem disease. PD likely starts due to a complex interaction of environmental toxins, susceptible genes, and advanced aging. PD has a larger arsenal of treatment choices than other neurodegenerative diseases. However, like the other neurodegenerative disorders, a cure for PD has not been reported. Thus, assembling this vast amount of information about PD feels like solving a complex puzzle. We close this work with Figure 7, which presents four pieces of the puzzle that could help in finding a solution to PD.

## Figures and Tables

**Table 1:** Neurobiochemical Pathogenesis of Parkinson’s Disease.

Relationship Equation	Pathogenic Effect and Putative Mechanism	Associated Clinical Features
Dopamine **↓**	Progressive degeneration and loss of dopaminergic neurons in the substantia nigra (corpus striatum), leading to a collapse of facilitatory motor control.	Primary motor deficits: Bradykinesia, akinesia, lead-pipe muscle rigidity.
Dopamine **↓** and Acetylcholine **↑**	Disruption of physiological balance in the corpus striatum. The lack of inhibitory dopamine causes a relative or absolute hyperactivity of excitatory cholinergic pathways.	Additional motor deficits: Resting tremor and progressive fine motor or cognitive memory deficits.
Glutamate and GABA Dysfunction	Imbalance between excitatory (glutamatergic) and inhibitory (GABAergic) signaling within the regulatory circuits of the basal ganglia, inducing excitotoxic hyperactivity.	Global motor circuit dysfunction: Incoordination, postural instability, and motor blocks (Freezing of gait).
Serotonin **↓**	Impairment and loss of serotonergic neurons within the raphe nuclei, which are responsible for modulating the limbic circuits and circadian rhythms.	Neuropsychiatric and vegetative symptoms: Depression, anxiety, sleep fragmentation, and sleep disturbances.
Noradrenaline **↓**	Early degeneration of neurons in the locus coeruleus resulting in a deficit of attentional modulation and impaired control of the autonomic nervous system.	Cognitive and autonomic deficits: Dysautonomia (e.g., orthostatic hypotension) and marked reduction in attention span.

The ‘down arrow’ represents a reduction in neurotransmitter, whereas the ‘up arrow’ represents an increase in neurotransmitter.

## Data Availability

No new data were created or analyzed in this study.

## Supplementary Materials

The following supporting information can be downloaded at: https://www.mdpi.com/article/10.3390/neurosci7040093/s1, Table S1. Motor and Non-Motor Symptoms of Parkinson’s Disease *. Table S2. Medications for Treating the Motor Symptoms of PD #. Table S3. Medications for Managing the Non-Motor Symptoms of Excessive Drooling, Orthostatic Hypotension, Gastrointestinal Symptoms and Bladder Control in PD *. Table S4. Medications for Managing the Non-Motor Symptoms of Anxiety and Depression in PD * 4. Table S5. Medications for Managing the Non-Motor Symptoms of Dementia and Psychosis in PD *.
