# Supplementary material for: Parkinson’s Disease: Pathophysiology, Treatment Strategies, Wellness Approaches, and Obstacles/Paths Forward
Source: NeuroSci. 2026 Aug 21;7(4):93. doi: 10.3390/neurosci7040093 (PMC13516459; doi:10.3390/neurosci7040093)
Supplement: Supplementary file 1 [file neurosci-07-00093-s001.zip › neurosci-4512635-supplementary.pdf]

NeuroSci

MDPI

Supplemental Material to:
Parkinson's Disease: Pathophysiology, Treatment Strategies, Wellness Approaches, and Obstacles/Paths Forward

Frank C. Church, Jill C. Rau, and Stefania Brotini

Table S1. Motor and Non-Motor Symptoms of Parkinson's Disease*

Motor-Related Symptoms	% Occurrence	(Reference)
"Cardinal" Signs		
Bradykinesia (Slowed movement)	90%	[1]
Postural Instability (Difficulty staying balanced)	20%	[2]
Resting Tremor (Involuntary shaking)	70%	[3]
Rigidity (Resistance to movement)	90%	[4]
Other		
Dystonia (Abnormal muscle contractions)	30%	[5]
Facial Masking (Hypomimia)	50-70%	[6]
Freezing of Gait (FOG)	52-86%	[7]
Gait Disturbances (Change in walking)	7-60%	[8]
Handwriting Changes (Micrographia)	50-75%	[9]
Levodopa-induced Dyskinesia (Uncontrolled movement)	9-80%	[10]
Speech Problems (Dysarthria, hypophonia)	75-90%	[11]
**Non-Motor-Related Symptoms**	**% Occurrence**	**(Reference)**
Autonomic Nervous System Disorder		
Cardiovascular		
Orthostatic Hypotension	30-40%	[12]
Gastrointestinal		
Constipation	5-80%	[13]
Dysphagia (Difficulty swallowing)	35%	[14]
Sialorrhea	10-84%	[15]
Sexual		
Sexual Dysfunction	68% male/53% female	[16]
Urinary		
Bladder Dysfunction	50%	[17]
Chronic Pain	30-50%	[18]
Cognitive Impairment		
Executive function impairment	20-40%	[19]
Mild cognitive impairment	25-30%	[20]
PD dementia	90%	[21]
Subjective cognitive decline	28%	[22]
Neuropsychiatric Manifestations		
Anxiety	20-44%	[23]
Apathy	40%	[24]
Depression	40-55%	[25]
Fatigue	33-80%	[26]
Psychosis	20-79%	[27]
Olfactory Dysfunction	90%	[28]
Sleep Disorders		

Circadian dysfunction	60%	[29]
Excessive daytime sleepiness	55%	[30]
Insomnia	60%	[31]
Obstructive sleep apnea in PD	45%	[32]
REM Sleep Behavior Disorder (RBD)	25-50%	[33]
Restless leg syndrome in PD	19%	[34]
Visual/Visuospatial functioning	87%	[35]

*The table is partly based on [36-42].

Table S2. Medications for Treating the Motor Symptoms of PD#.

**Levodopa**				
**Brand Name**	**Formulation**	**Most common side effects**	**Use in treating disease**	**Additional comments**
Sinemet Immediate-release (IR) carbidopa/levodopa tablets	10/100, 25/100 or 25/250 mg/mg	Nausea, low blood pressure dizziness, confusion, and dyskinesia	For treating motor symptoms, e.g., stiffness, and tremor	Usually starts in 45-60 min, do not take with protein
Dhivy Immediate-release carbidopa/levodopa tablets	25/100 mg/mg	Same as above	Same as above	Can be broken into ¼ or ½ size if needed;
Sinemet CR- Controlled-release (CR) carbidopa/levodopa tablets*	25/100 or 50/250 mg/mg	Same as above	Same as above	May not provide enough symptom relief for most PwP
*Rytary Extended-release (ER) carbidopa/levodopa capsules***	23.75/95, 36.25/145, 48.75/195, or 61.25/245 mg/mg	Same as above	Same as above	Lasts longer than IR and CR forms of Sinemet
*Crexont Extended-release (ER) carbidopa/levodopa capsules***	35/140, 52.5/210, 70/280, 87.50/350 mg/mg	Same as above	Same as above	Lasts longer than Rytary, do not open capsule, do not take with protein
Stalevo Carbidopa/Levodopa/Entacapone tablets	12.5/50/200, 18.75/75/200, 25/100/200, 31.25/150/200, or 50/200/200	Nausea, low blood pressure, dizziness, dyskinesia, diarrhea, and behavioral issues	Replacement for IR carbidopa/levodopa, off/on timing issue	3 drugs in one tablet with carbidopa/levodopa/entacapone in a single tablet
Duopa- Enteral suspension cassette of carbidopa/levodopa	4.63 mg carbidopa/20 mg levodopa/mL	Same as above	Useful for off/on timing and usually reserved for advanced PD	Continuous infusion pumped to the small intestine
Inbrija Levodopa inhalation powder capsule	42 mg	Same as above but add cough	Used to help sudden off period and used with carbidopa/levodopa	Bypasses GI tract, and may help those with constipation
Vyalev Foscarbidopa/Foslevodopa for subcutaneous infusion	120 mg Foscarbidopa and 2,400 mg foslevodopa / 10 mL mixture	Nausea, dyskinesia, site irritation, blood pressure drop, vomiting, confusion, hallucinations	Used in advanced disease especially those with off periods when symptoms become worse	Continuous infusion pumped into belly region using a small pump
**Dopamine Agonists (DA)**				
**Brand Name**	**Formulation**	**Most common side effects**	**Use in treating disease**	**Additional comments**
Requip Ropinirole tablets*	0.25, 0.5, 1, 2, 3, or 5 mg	Nausea, dizziness, leg swelling, confusion, and behavioral changes with compulsive behavior	For treating motor symptoms, e.g., stiffness, slowness, and tremor	For many PwP, a DAs were used before starting carbidopa/levodopa
Mirapex Pramipexole tablets	0.125, 0.25, 0.5, 0.75, 1.0, or 1.5 mg	Same as above	Same as above	Is longer lasting than carbidopa/levodopa
Neupro Rotigotine transdermal patch	1, 2, 3, 4, 6, or 8 mg	Same as above but add skin irritation	Same as above	Same as above, and bypasses GI tract

Apokyn Apomorphine hydrochloride subcutaneous injection	30 mg in 3 mL vial	Like other dopamine agonists above but add redness or bruising at the injection site	Useful as a rescue or for off episodes with severe symptoms	Advised to use anti-nausea medication as needed
Kynmobi Apomorphine hydrochloride sublingual film	10, 15, 20, 25, or 30 mg containing film	Nausea and vomiting, orthostatic hypotension, interaction with serotonin antagonists, and behavioral changes	First drug treatment for off episodes of carbidopa/levodopa	No longer available in the USA/Canada but is available in Europe
**Monoamine oxidase B (MAO-B) inhibitors**				
**Brand Name**	**Formulation**	**Most common side effects**	**Use in treating disease**	**Additional comments**
Azilect Rasagiline Tablets	0.5 or 1 mg	Nausea, dry mouth, dizziness, and constipation	For increasing availability of levodopa	Can cause jitteriness, confusion or sleep issues
Eldepryl or L-Deprenyl Selegiline Tablets	5 or 10 mg	Same as above	Same as above	Same as above
Xadago Safinamide Tablets	50 or 100 mg	Same as above	Same as above	Same as above
**Catechol-O-methyl transferase (COMT) inhibitors**				
**Brand Name**	**Formulation**	**Most common side effects**	**Use in treating disease**	**Additional comments**
Comtan Entacapone tablets	200 mg	Increased side effects of Carbidopa/levodopa, and severe diarrhea	Sustained Levodopa, decreases off time drop off period	Urine and other body fluids turn reddish-brown color
Ongentys Opicapone tablets	25 or 50 mg	Increased side effects of Carbidopa/levodopa	Same as above	Avoid food one hour before or later
Tasmar Talcapone tablets	100 mg	Same as above, but with increased diarrhea and liver damage	Same as above	Rarely used because of serious side effects
**Amantadine**				
**Brand Name**	**Formulation**	**Most common side effects**	**Use in treating disease**	**Additional comments**
Symmetrel Amantadine capsules*	100 mg	Nausea, insomnia, confusion, lightheaded, swollen feet, dry mouth, hallucinations	To combat levodopa-induced dyskinesia, and other symptoms	Can cause a purplish discoloration of the skin on legs
Osmolex ER Extended-release amantadine tablets	129, 193, or 258 mg	Same as above	Same as above	ER medications should not be crushed
Gocovri extended-release amantadine capsules	68.5 or 137 mg	Same as above	Same as above, and used to treat off-time issues	Same as above
**Anticholinergics**				
**Brand Name**	**Formulation**	**Most common side effects**	**Use in treating disease**	**Additional comments**

Artane Trihexyphenidyl tablets*	2 or 5 mg	Confusion, drowsiness, memory. Issues, hallucinations, dry mouth, increased heart rate, dizziness, urinary retention	For tremor and dystonia, used in Young Onset PD	Avoid use in older adults
Cogentin Benztropine tablets	0.5, 1, or 2 mg	Same as above	Same as above	Same as above

5*Drug formerly known by this name. **Extended-release (ER) capsules are not interchangeable with other carbidopa/levodopa products
#The table is partly based on the following [43-49].

Table S3. Medications for Managing the Non-Motor Symptoms of Excessive Drooling, Orthostatic Hypotension, Gastrointestinal Symptoms and Bladder Control in PD*.

Excessive Drooling

Medication	Brand Names	Formulation	Most common side effects	Use in treating disease
Atropine Drops		1% ophthalmic solution	Dry mouth	Excessive drooling
Botulinum toxin A	Xeomin	50, 100, or 200 unit vial	Same as above	Same as above
Botulinum toxin B	Myobloc	2,500 units/0.5 mL<br>5,000 units/mL	Same as above	Same as above
Glycopyrrolate	Glycate, Cuvposa	Glycate- 1, 1.5, or 2 mg tablets<br>Cuvposa- 1 mg/mL solution	Dry mouth, drowsiness, convulsions, nausea, vomiting, dizziness, blurred vision, constipation, headache, and urinary retention	Same as above
Scopolamine	Transderm Scop	1.5 mg patch	Same as above	Same as above
#Biperiden hydrochloride	Akineton	2 mg tablet	Dry mouth; blurred vision; drowsiness; euphoria or disorientation; urinary retention; postural hypotension, constipation; agitation; disturbed behavior	Same as above

Constipation

Medication	Brand Names	Formulation	Most common side effects	Use in treating disease
Lubipostone	Amitiza	8 or 24 mcg capsules	Bloating, gas, upset stomach, dizziness, and chest pain	Constipation
Polyethylene glycol 3500	MiraLax (OTC)	1 capful (17 gm) in 4-8 oz water	Bloating, gas, diarrhea, nausea, abdominal pain	Same as above
Linaclotide	Linzess	72 or 145 mcg capsules	Diarrhea, abdominal pain, gas, and headache	Chronic idiopathic constipation

Orthostatic Hypotension

Medication	Brand Names	Formulation	Most common side effects	Use in treating disease
Droxidopa	Northera	100, 200, or 300 mg capsules	Risk of high blood pressure when lying flat, nausea, headache and dizziness	Orthostatic hypotension
Fludrocortisone	Florinef	0.1 mg tablets	Leg swelling, low potassium, and risk of high blood pressure when lying flat	Same as above
Midodrine	ProAmatine	2.5, 5, or 10 mg tablets	Itchy scalp, urinary retention, risk of high blood pressure when lying flat, and headache	Same as above
Octreotide	Sandostatin	1 ampule of 1ml of 50, 100 or 500 mcg/ml	Stomach pain, nausea, and pain at the injection site, gallbladder problems, diarrhea, low heart rate, high blood sugar, and low thyroid hormone	Same as above
Pyridostigmine	Mestinon	30 or 60 mg tablets<br>60mg/5ml oral solution	Diarrhea, increased sweating, increased watering of mouth,	Same as above

			nausea or vomiting, or stomach cramps or pain	

Bladder Control – Antimuscarinics/Anticholinergics

Medication	Brand Names	Formulation	Most common side effects	Use in treating disease
Oxybutynin	Ditropan<br>Ditropan XL<br><br>Oxytrol	5 mg tablets<br>5, 10, or 15 mg ER tablets<br>3.9 mg/24 h patch	Dizziness, drowsiness, dry mouth, constipation, nausea, confusion	Overactive bladder
Solifenacin	Vesicare	5 or 10 mg tablets	Dry mouth, constipation, and dry eyes	Same as above
Tolterodine	Detrol<br>Detrol LA	1 or 2 mg tablets<br>2 or 3.4 mg ER tablets	Dry mouth, constipation, dizziness	Same as above

Bladder Control –Beta-3 Agonists

Medication	Brand Names	Formulation	Most common side effects	Use in treating disease
Mirabegran	Myrbetriq	25 or 50 mg ER tablets,	High blood pressure and headache	Same as above
Vibegron	Gemtesa	75 mg tablet	Headache, nasopharyngitis, Diarrhea, nausea, upper respiratory infection	Same as above

*The table is partly based on [43-49]. #In Europe, Akineton is also used for treating tremor. Furthermore, Akineton is no longer actively marketed or sold as a brand-name drug in the United States.

Table S4. Medications for Managing the Non-Motor Symptoms of Anxiety and Depression in PD*4

Benzodiazepines

Medication	Brand Names	Formulation	Most common side effects	Use in treating disease
Alprazolam	Xanax, Xanax XR	0.25, 0.5, 1, 2, or 3 mg tablets XR: 0.5, 1, 2, or 3	Drowsiness, tiredness, lightheaded nervousness, depression, headache, confusion dizziness, blurred vision	Anxiety
Clonazepam	Klonopin	0.5, 1, or 2 mg tablets and 0.125, 0.25, 0.5, 1, or 2 mg oral disinter-grating tablets	Same as above	Same as above
Diazepam	Valium	2 5, or 10 mg tablets and 5 mg/5 ml oral solution	Same as above	Same as above
Lorazepam	Ativan	0.5, 1, or 2 mg tablets and 2 mg/ml oral solution	Same as above	Same as above

Selective Serotonin Reuptake Inhibitors (SSRIs)

Medication	Brand Names	Formulation	Most common side effects	Use in treating disease
Fluoxetine	Prozac	10, 20 or 40 mg tablets 10, 20, or 40 mg capsules 20 mg/5mL solution	Headache, nausea, diarrhea, insomnia, dry mouth, sweating, jitteriness, decreased sex drive, weight gain or loss	Depression and anxiety
Sertraline	Zoloft	25, 50, or 100 mg tablets 20 mg/mL solution	Same as above	Same as above
Citalopram	Celexa	10, 20 or 40 mg tablets 10 mg/2 mL solution	Same as above	Same as above
Escitalopram	Lexapro	5, 10, or 20 mg tablets	Same as above	Same as above
Paroxetine	Paxil	10, 20, 30, or 40 mg tablets	Same as above	Same as above
	Paxil CR	25 mg tablet	Same as above	Same as above

Serotonin/Norepinephrine Reuptake Inhibitors (SNRIs)

Medication	Brand Names	Formulation	Most common side effects	Use in treating disease
Desvenlafaxin	Pristiq	25, 50, or 100 mg tablets	Nausea, dry mouth, dry mouth, dizziness, excessive sweating, constipation, trouble sleeping, decreased sex drive	Depression and anxiety
Duloxetine	Cymbalta	20, 30, 40, or 60 mg tablets	Same as above, plus may create liver problems	Same as above
Venlafaxine	Effexor, Effexor XR	25, 37.5, 50, 75 or 100 mg tablets; XR: 37.5, 50, 75, 150, or 225 mg tablets	Same as above, plus it may increase blood pressure	Same as above

Medication	Brand Names	Formulation	Most common side effects	Use in treating disease
**Tricyclic compounds**				
Amitriptyline	Elavil	10, 25, 50, 75, 100 or 150 mg tablets	Dry mouth, dizziness, drowsiness, headache, blurred vision, confusion, constipation, nausea, urinary retention	Depression and anxiety
Nortriptyline	Palmelor	10, 25, 50, 75, 100, 150 mg capsules, and 10 mg/5 mL solution	Same as above	Same as above
Desipramine	Norpramin	10, 25, 50, 75, 100, and 150 mg tablets	Same as above	Same as above
**Other Anti-anxiety**				
Buspione	BuSpar	5, 7.5, 10, 15, or 30 tablets	Dizziness, drowsiness, dry mouth, nausea, and headache	Anxiety
Propranolol	Inderal	10, 20, 40, 60 or 80 mg tablets and 20 or 40 mg/5 mL	Decreased heart rate, depression, asthma	Same as above
**Other Antidepressants**				
Mirtazapine	Remeron SolTabs	7.5, 15, 30, or 45 mg SolTabs: 15, 30, or 45 mg Oral disintegrating tabs	Drowsiness, dizziness, increased - appetite, weight gain, constipation, vivid dreams	Depression and anxiety
Bupropion	Wellbutrin, SR and XL	75 or 100 mg tablets SR: 100, 150, or 200 mg XL: 150 or 300 mg tablets	Tiredness, dry mouth, drowsiness, lightheaded, depression, headache, confusion, dizziness, weight loss	Same as above

*The table is partly based on [43,44,47-49].

Table S5. Medications for Managing the Non-Motor Symptoms of Dementia and Psychosis in PD*

**Dementia**				
**Medication**	**Brand Names**	**Formulation**	**Most common side effects**	**Use in treating disease**
Donepezil	Aricept,<br>Aricept ODT	5, 10, or 23 mg tablets<br>5 or 10 mg oral self-disintegrating	Nausea, headache, diarrhea, vomiting, insomnia, dizziness, muscle cramps and slow heart rate	Dementia<br>Same as above
Rivastigmine	Exelon	1.5, 3, 4.5 or 6 mg capsules<br>4.6, 8.5, or 13.3 mg/24 hour patch	Same as above	Same as above
Galantamine	Razadyne,<br>Razadyne ER	4, 8, or 12 mg tablets, or<br>4 mg/ml oral solution,	Same as above	Same as above
Memantine	Namendo<br>Namendo XR	5 or 10 mg tablets<br>2 mg/mL oral solution<br>7, 14, 21 or 28 mg tablets	Dizziness, headache, constipation, confusion	Same as above

**Psychosis**				
**Medication**	**Brand Names**	**Formulation**	**Most common side effects**	**Use in treating disease**
Clozapine	Clorazil<br>FazaClo<br>Versacloz	Clorazil: 25, 50, 100, or 200 mg tablet; FazaClo: 12.5, 25, 10, 150 or 200 mg orally disintegrating tablets; Versacloz: 50 mg/ mL oral suspension	Drowsiness, drooling, tachycardia, dizziness, constipation, low blood pressure, headache; blood test for white blood cell count is important	Psychosis (hallucinations and delusions)
Pimavanserin	Nuplazid	34 mg capsules	Nausea, swelling of limbs, confusion	Same as above
Quetiapine	Seroquel,<br>Seroquel XR	25, 50, 100, 150, 200, 300, or 400 mg tablets; XR: 50, 150, 200, 300 or 400 mg tablets	Sleepiness, dry mouth, dizziness, low blood pressure, stomach pain, weight gain	Same as above

*The table is partly based on [43-49]

References

1. Gasser, T.; Wichmann, T. Chapter 16 - Parkinson disease and other synucleinopathies**Thoughts on teaching about the neurobiology of diseases section available at the beginning of the book and Index terms are available at the end of the book. In *Neurobiology of Brain Disorders (Second Edition)*, Zigmond, M.J., Wiley, C.A., Chesselet, M.-F., Eds.; Academic Press: 2023; pp. 253-274.
2. Bath, J.E.; Wang, D.D. Unraveling the threads of stability: A review of the neurophysiology of postural control in Parkinson's disease. *Neurotherapeutics* **2024**, *21*, e00354, doi:https://doi.org/10.1016/j.neurot.2024.e00354.

3. Dirkx, M.F.; Bologna, M. The pathophysiology of Parkinson's disease tremor. *Journal of the Neurological Sciences* **2022**, *435*, 120196, doi:https://doi.org/10.1016/j.jns.2022.120196.
4. Asci, F.; Falletti, M.; Zampogna, A.; Patera, M.; Hallett, M.; Rothwell, J.; Suppa, A. Rigidity in Parkinson's disease: evidence from biomechanical and neurophysiological measures. *Brain* **2023**, *146*, 3705-3718, doi:10.1093/brain/awad114.
5. Kidron, D.; Melamed, E. Forms of dystonia in patients with Parkinson's disease. *Neurology* **1987**, *37*, 1009-1011, doi:10.1212/wnl.37.6.1009.
6. Marsili, L.; Agostino, R.; Bologna, M.; Belvisi, D.; Palma, A.; Fabbrini, G.; Berardelli, A. Bradykinesia of posed smiling and voluntary movement of the lower face in Parkinson's disease. *Parkinsonism & related disorders* **2014**, *20*, 370-375.
7. Giladi, N.; Tal, J.; Azulay, T.; Rascol, O.; Brooks, D.J.; Melamed, E.; Oertel, W.; Poewe, W.H.; Stocchi, F.; Tolosa, E. Validation of the freezing of gait questionnaire in patients with Parkinson's disease. *Movement disorders: official journal of the Movement Disorder Society* **2009**, *24*, 655-661.
8. Chen, P.-H.; Wang, R.-L.; Liou, D.-J.; Shaw, J.-S. Gait Disorders in Parkinson's Disease: Assessment and Management. *International Journal of Gerontology* **2013**, *7*, 189-193, doi:https://doi.org/10.1016/j.ijge.2013.03.005.
9. Thomas, M.; Lenka, A.; Kumar Pal, P. Handwriting Analysis in Parkinson's Disease: Current Status and Future Directions. *Mov Disord Clin Pract* **2017**, *4*, 806-818, doi:10.1002/mdc3.12552.
10. Thanvi, B.; Lo, N.; Robinson, T. Levodopa-induced dyskinesia in Parkinson's disease: clinical features, pathogenesis, prevention and treatment. *Postgrad Med J* **2007**, *83*, 384-388, doi:10.1136/pgmj.2006.054759.
11. Dashtipour, K.; Tafreshi, A.; Lee, J.; Crawley, B. Speech disorders in Parkinson's disease: pathophysiology, medical management and surgical approaches. *Neurodegener Dis Manag* **2018**, *8*, 337-348, doi:10.2217/nmt-2018-0021.
12. Velseboer, D.C.; de Haan, R.J.; Wieling, W.; Goldstein, D.S.; de Bie, R.M. Prevalence of orthostatic hypotension in Parkinson's disease: a systematic review and meta-analysis. *Parkinsonism Relat Disord* **2011**, *17*, 724-729, doi:10.1016/j.parkreldis.2011.04.016.
13. Sauerbier, A.; Ray Chaudhuri, K. Non-motor symptoms: the core of multi-morbid Parkinson's disease. *Br J Hosp Med (Lond)* **2014**, *75*, 18-24, doi:10.12968/hmed.2014.75.1.18.
14. Kalf, J.G.; de Swart, B.J.M.; Bloem, B.R.; Munneke, M. Prevalence of oropharyngeal dysphagia in Parkinson’s disease: A meta-analysis. *Parkinsonism & Related Disorders* **2012**, *18*, 311-315, doi:https://doi.org/10.1016/j.parkreldis.2011.11.006.
15. Ou, R.; Guo, X.; Wei, Q.; Cao, B.; Yang, J.; Song, W.; Chen, K.; Zhao, B.; Chen, X.; Shang, H. Diurnal drooling in Chinese patients with Parkinson's disease. *J Neurol Sci* **2015**, *353*, 74-78, doi:10.1016/j.jns.2015.04.007.
16. Raciti, L.; De Cola, M.C.; Ortelli, P.; Corallo, F.; Lo Buono, V.; Morini, E.; Quattrini, F.; Filoni, S.; Calabrò, R.S. Sexual Dysfunction in Parkinson Disease: A Multicenter Italian Cross-sectional Study on a Still Overlooked Problem. *J Sex Med* **2020**, *17*, 1914-1925, doi:10.1016/j.jsxm.2020.06.010.
17. Verbaan, D.; Marinus, J.; Visser, M.; van Rooden, S.M.; Stiggelbout, A.M.; van Hilten, J.J. Patient-reported autonomic symptoms in Parkinson disease. *Neurology* **2007**, *69*, 333-341, doi:10.1212/01.wnl.0000266593.50534.e8.
18. Fil, A.; Cano-de-la-Cuerda, R.; Muñoz-Hellín, E.; Vela, L.; Ramiro-González, M.; Fernández-de-Las-Peñas, C. Pain in Parkinson disease: a review of the literature. *Parkinsonism Relat Disord* **2013**, *19*, 285-294; discussion 285, doi:10.1016/j.parkreldis.2012.11.009.
19. Dirnberger, G.; Jahanshahi, M. Executive dysfunction in Parkinson's disease: a review. *J Neuropsychol* **2013**, *7*, 193-224, doi:10.1111/jnp.12028.
20. Weintraub, D.; Tröster, A.I.; Marras, C.; Stebbins, G. Initial cognitive changes in Parkinson's disease. *Mov Disord* **2018**, *33*, 511-519, doi:10.1002/mds.27330.
21. Gratwicke, J.; Jahanshahi, M.; Foltynie, T. Parkinson's disease dementia: a neural networks perspective. *Brain* **2015**, *138*, 1454-1476, doi:10.1093/brain/awv104.
22. Yang, N.; Ju, Y.; Ren, J.; Wang, H.; Li, P.; Ning, H.; Tao, J.; Liu, W. Prevalence and affective correlates of subjective cognitive decline in patients with de novo Parkinson's disease. *Acta Neurol Scand* **2022**, *146*, 276-282, doi:10.1111/ane.13662.

23. Upneja, A.; Paul, B.S.; Jain, D.; Choudhary, R.; Paul, G. Anxiety in Parkinson's Disease: Correlation with Depression and Quality of Life. *J Neurosci Rural Pract* **2021**, *12*, 323-328, doi:10.1055/s-0041-1722840.
24. den Brok, M.G.; van Dalen, J.W.; van Gool, W.A.; Moll van Charante, E.P.; de Bie, R.M.; Richard, E. Apathy in Parkinson's disease: A systematic review and meta-analysis. *Mov Disord* **2015**, *30*, 759-769, doi:10.1002/mds.26208.
25. Cong, S.; Xiang, C.; Zhang, S.; Zhang, T.; Wang, H.; Cong, S. Prevalence and clinical aspects of depression in Parkinson's disease: A systematic review and meta-analysis of 129 studies. *Neurosci Biobehav Rev* **2022**, *141*, 104749, doi:10.1016/j.neubiorev.2022.104749.
26. Stocchi, F.; Abbruzzese, G.; Ceravolo, R.; Cortelli, P.; D'Amelio, M.; De Pandis, M.F.; Fabbrini, G.; Pacchetti, C.; Pezzoli, G.; Tessitore, A.; et al. Prevalence of fatigue in Parkinson disease and its clinical correlates. *Neurology* **2014**, *83*, 215-220, doi:10.1212/wnl.0000000000000587.
27. Levin, J.; Hasan, A.; Höglinger, G.U. Psychosis in Parkinson's disease: identification, prevention and treatment. *J Neural Transm (Vienna)* **2016**, *123*, 45-50, doi:10.1007/s00702-015-1400-x.
28. Doty, R.L. Olfactory dysfunction in Parkinson disease. *Nat Rev Neurol* **2012**, *8*, 329-339, doi:10.1038/nrneurol.2012.80.
29. Videnovic, A.; Noble, C.; Reid, K.J.; Peng, J.; Turek, F.W.; Marconi, A.; Rademaker, A.W.; Simuni, T.; Zadikoff, C.; Zee, P.C. Circadian Melatonin Rhythm and Excessive Daytime Sleepiness in Parkinson Disease. *JAMA Neurology* **2014**, *71*, 463-469, doi:10.1001/jamaneurol.2013.6239.
30. Chahine, L.M.; Amara, A.W.; Videnovic, A. A systematic review of the literature on disorders of sleep and wakefulness in Parkinson's disease from 2005 to 2015. *Sleep Med Rev* **2017**, *35*, 33-50, doi:10.1016/j.smrv.2016.08.001.
31. Sobreira-Neto, M.A.; Pena-Pereira, M.A.; Sobreira, E.S.T.; Chagas, M.H.N.; de Almeida, C.M.O.; Fernandes, R.M.F.; Tumas, V.; Eckeli, A.L. Chronic Insomnia in Patients With Parkinson Disease: Which Associated Factors Are Relevant? *J Geriatr Psychiatry Neurol* **2020**, *33*, 22-27, doi:10.1177/0891988719856687.
32. Aini, N.; Putri, A.R.; Banda, K.J.; Janitra, F.E. Meta-analysis of the prevalence of obstructive sleep apnea and associated risk factors among Parkinson’s disease. *Sleep and Biological Rhythms* **2025**, *23*, 101-112, doi:10.1007/s41105-024-00561-w.
33. Iranzo, A.; De Cock, V.C.; Fantini, M.L.; Pérez-Carbonell, L.; Trotti, L.M. Sleep and sleep disorders in people with Parkinson's disease. *The Lancet Neurology* **2024**, *23*, 925-937.
34. Peeraully, T.; Tan, E.K. Linking restless legs syndrome with Parkinson's disease: clinical, imaging and genetic evidence. *Transl Neurodegener* **2012**, *1*, 6, doi:10.1186/2047-9158-1-6.
35. Davidsdottir, S.; Cronin-Golomb, A.; Lee, A. Visual and spatial symptoms in Parkinson’s disease. *Vision Research* **2005**, *45*, 1285-1296, doi:https://doi.org/10.1016/j.visres.2004.11.006.
36. Silva, A.B.R.L.; de Oliveira, R.W.G.; Diógenes, G.P.; de Castro Aguiar, M.F.; Sallem, C.C.; Lima, M.P.P.; de Albuquerque Filho, L.B.; de Medeiros, S.D.P.; de Mendonça, L.L.P.; de Santiago Filho, P.C. Premotor, nonmotor and motor symptoms of Parkinson's disease: a new clinical state of the art. *Ageing research reviews* **2023**, *84*, 101834.
37. Berganzo, K.; Tijero, B.; Gonzalez-Eizaguirre, A.; Somme, J.; Lezcano, E.; Gabilondo, I.; Fernandez, M.; Zarranz, J.; Gómez-Esteban, J. Motor and non-motor symptoms of Parkinson's disease and their impact on quality of life and on different clinical subgroups. *Neurología (English Edition)* **2016**, *31*, 585-591.
38. Lee, H.M.; Koh, S.-B. Many faces of Parkinson’s disease: non-motor symptoms of Parkinson’s disease. *Journal of movement disorders* **2015**, *8*, 92.
39. Vuletić, V. Non-motor Symptoms in Parkinson’s Disease. *Mind and Brain* **2020**, 109-118, doi:10.1007/978-3-030-38606-1
40. Schapira, A.H.; Chaudhuri, K.R.; Jenner, P. Non-motor features of Parkinson disease. *Nature Reviews Neuroscience* **2017**, *18*, 435.
41. Jankovic, J. Motor fluctuations and dyskinesias in Parkinson's disease: clinical manifestations. *Mov Disord* **2005**, *20*, doi:10.1002/mds.20458.
42. Moustafa, A.A.; Chakravarthy, S.; Phillips, J.R.; Gupta, A.; Keri, S.; Polner, B.; Frank, M.J.; Jahanshahi, M. Motor symptoms in Parkinson’s disease: A unified framework. *Neuroscience & Biobehavioral Reviews* **2016**, *68*, 727-740.

43. Hauser, R.A.; Torres-Yaghi, Y.; Fisher, S.; Banisadr, G. How to dose extended-release carbidopa-levodopa capsules (IPX203, CREXONT®) in patients with Parkinson's disease. *Clinical Parkinsonism & Related Disorders* **2025**, 100357.
44. Kobylecki, C. Update on the diagnosis and management of Parkinson's disease. *Clinical Medicine* **2020**, *20*, 393-398.
45. Rizek, P.; Kumar, N.; Jog, M.S. An update on the diagnosis and treatment of Parkinson disease. *Cmaj* **2016**, *188*, 1157-1165.
46. Rawls, A.; Okun, M.S. Parkinson Disease. *CONTINUUM: Lifelong Learning in Neurology* **2025**, *31*, 930-955.
47. Tanner, C.M.; Ostrem, J.L. Parkinson's Disease. *N. Engl. J. Med.* **2024**, *391*, 442-452, doi:https://doi.org/10.1056/NEJMra2401857.
48. Church, F.C. Treatment Options for Motor and Non-Motor Symptoms of Parkinson's Disease. *Biomolecules* **2021**, *11*, 612.
49. Pahwa, R.; Peron, E.; Cloud, L. Medications: A Treatment Guide to Parkinson's Disease *https://www.parkinson.org/library/books/medications* **2025**, *Parkinson's Foundation Book*.
